# Construction, evaluation, and applications of renal barrier-on-a-chip system

**DOI:** 10.1016/j.bioactmat.2025.12.032

**Published:** 2026-01-02

**Authors:** Tuya Naren, Weikang Lv, Abdellah Aazmi, Yujun Wang, Haoran Yu, Jie Ying Lee, Huixiang Yang, Mengfei Yu, Xiuxiu Jiang, Huayong Yang, Liang Ma

**Affiliations:** a State Key Laboratory of Fluid Power and Mechatronic Systems, Zhejiang University, Hangzhou, 310058, China; b School of Mechanical Engineering, Zhejiang University, Hangzhou, 310058, China; c The Affiliated Stomatologic Hospital, School of Medicine, Zhejiang University, Hangzhou, 310003, China; d Zhejiang Provincial Clinical Research Center for Gynecology, Zhejiang Key Laboratory of Maternal and Infant Health, Department of Family Planning, Women's Hospital, Zhejiang University School of Medicine, Hangzhou City, Zhejiang Province, 310006, China

**Keywords:** Kidney-on-a-chip, Glomerular filtration barrier, Proximal tubule, Microphysiological systems, Drug nephrotoxicity

## Abstract

Organ-on-a-chip (OoC) technology offers a transformative approach to modeling the human renal barrier, overcoming limitations of traditional animal and two-dimensional cell models. This review systematically outlines the construction and evaluation of renal barrier biochips, focusing on the glomerular filtration barrier (GFB), tubular reabsorption barrier (TRB), and collecting duct regulatory barrier (CDRB). OoC platforms integrate biomimetic materials, simulate dynamic microenvironments, and use multicellular co-culture strategies. This enables them to closely replicate the structural and functional characteristics of renal barriers. Key evaluation metrics—including structural biomimicry, barrier integrity, and active transport functions—are discussed to validate model performance. The technology demonstrates significant potential in drug nephrotoxicity prediction, disease mechanism investigation, and regenerative medicine. Despite challenges in material properties and long-term functional maintenance, ongoing advancements in OoC design and integration are poised to enhance its application in precision medicine and kidney disease research.

## Highlights

• Presents a biomimetic renal barrier chip replicating glomerular, tubular, collecting duct structural-functional traits.
• Highlights dynamic microenvironment simulation (fluid shear stress, biochemical gradients) for high-fidelity renal modeling.
• Proposes a multidimensional framework validating structural/functional maturity of kidney-on-a-chip models.
• Demonstrates applications in predictive nephrotoxicity screening, personalized disease modeling, regenerative medicine.
• Discusses challenges and future directions: multi-organ integration, enhanced biomaterials, and clinical translation.

## Graphical abstract

**Figure:**
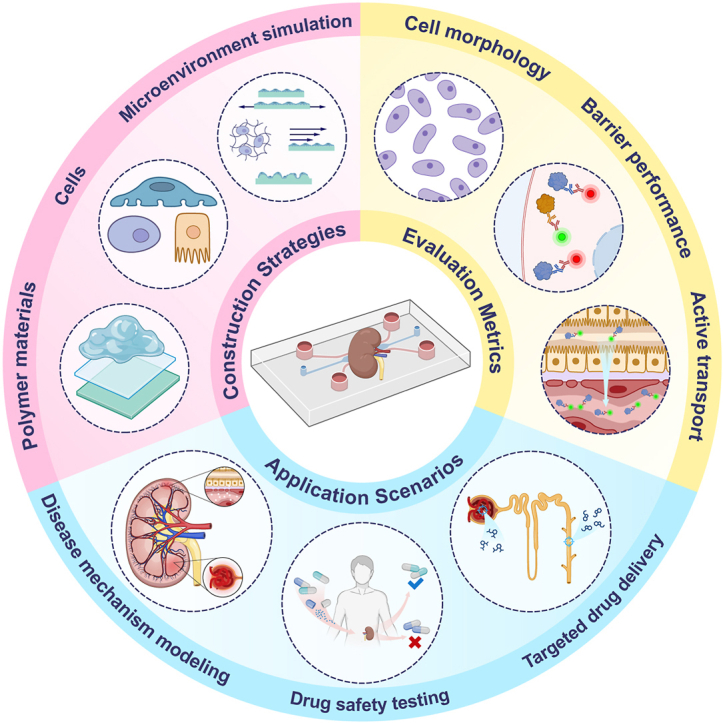

## 1 Introduction

The kidney is a core organ for maintaining homeostasis of the body's internal environment [1]. Its complex three-dimensional (3D) barrier system—comprising the GFB TRB, and CDRB—performs critical functions including metabolic waste clearance [2,3], ion balance regulation, and endocrine activity through sophisticated spatial organization and dynamic material exchange [[4], [5], [6]]. However, traditional research models, such as animal experiments and two-dimensional (2D) cell cultures, exhibit significant and inherent limitations [7]. Due to species differences, animal models fail to accurately reflect the unique pathological mechanisms and drug responses of the human kidney [8]. Conversely, static 2D cell models are entirely incapable of recapitulating key elements within the complex 3D microenvironment of the kidney [9,10]. These include hemodynamic shear stress, trans-barrier pressure gradients, 3D intercellular interactions, and dynamic substance exchange processes [[11], [12], [13]]. This fundamental disconnect directly leads to a significant disparity between preclinical data and actual human responses in drug development and disease research [[14], [15], [16], [17]].

The emergence of OoC technology offers a novel paradigm to address this longstanding challenge. The core of this technology lies in leveraging microfluidic systems, advanced biomaterials, and multicellular co-culture strategies to closely replicate critical features and complex functional units of organ tissues in vitro [10,18], such as thermal, mechanical forces, electrical, physicochemical cues, biochemical gradients, and intercellular communication [[19], [20], [21]]. Unlike organoids or conventional 3D cultures, OoCs uniquely integrate dynamic fluid flow, mechanical stimuli, and real-time monitoring capabilities [22]. These enable precise recapitulation of physiological filtration, reabsorption, and secretion processes—key functions that are often underdeveloped in self-assembled organoid systems [[23], [24], [25]]. For renal barrier research, OoC technology demonstrates unique potential: Glomerulus-on-a-chip models hold promise for recapitulating the molecular sieving and charge-selective functions of the GFB. This potential can be achieved through co-culture of endothelial cells and podocytes [2], coupled with precise regulation of transmembrane pressure [26,27]. Tubule-on-a-chip models can simulate the dynamics of substance transport during reabsorption and secretion processes by utilizing fluid shear stress and polarized culture environments within microchannels [6,9,28].

Recent advances in biomaterials, sensor integration, and patient-specific induced pluripotent stem cells (iPSCs) differentiation have further enhanced the physiological relevance and translational potential of renal OoCs. It is necessitating an updated systematic review that not only synthesizes these developments but also critically evaluates standardized evaluation frameworks and clinical translation pathways—aspects not comprehensively covered in earlier reviews.

**Fig. 1:**
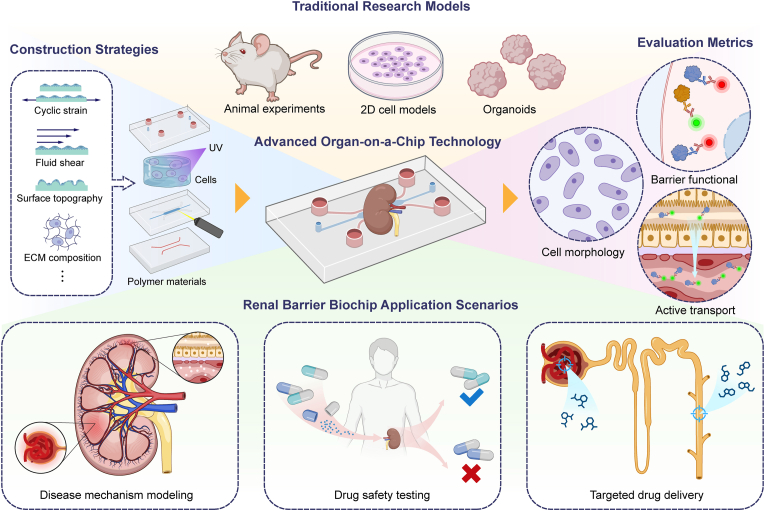
Schematic overview of the review framework on renal barrier-on-a-chip systems.

The primary objective of this review is to systematically elucidate how OoC technology can be employed to construct high-fidelity renal barrier models that overcome the limitations of traditional approaches. This is achieved through biomimetic design, dynamic microenvironment simulation, and functional integration. It begins by outlining the core biological characteristics of renal barriers and identifying the fundamental goals and challenges of their in vitro simulation. Subsequently, we critically analyze strategies for constructing renal barrier chips, with an in-depth focus on how material selection and fabrication, cell model choice, structural design, and microenvironment simulation. These enable accurate modeling of physiological and pathological barrier functions. A comprehensive evaluation framework encompassing multiple dimensions will be presented, emphasizing the inherent advantage of OoC platforms in enabling real-time and dynamic monitoring. Furthermore, we highlight the transformative applications and value of this technology in drug development and disease research. Finally, we address the current key technological bottlenecks and discuss prospective future directions (see Fig. 1).

Through this structured framework, this review aims to provide a valuable reference for gaining in-depth understanding of the principles, progress, and potential of renal barrier-on-a-chip technology, and to facilitate its translational application in kidney research, drug development, and precision medicine.

## 2 Biological basis of the renal barrier: core objectives and challenges for chip simulation

The kidney, as a core organ for maintaining homeostasis of the body's internal environment, relies critically on a sophisticated 3D barrier system for its function [29]. These barriers, primarily encompassing the GFB, TRB, and CDRB, using their unique spatial organization, cellular specialization, and dynamic substance exchange to achieve essential physiological processes [30,31], such as metabolic waste clearance, ion balance regulation, and endocrine activity [9,32,33]. An in-depth understanding is needed of both the structure and function of these barriers under healthy conditions, and the specific mechanisms underlying their disruption in pathological states. This understanding constitutes the essential foundation and prerequisite for constructing high-fidelity renal barrier OoC models. The core challenge for OoC technology lies precisely in how to accurately recapitulate these critical biological properties in an in vitro setting.

### 2.1 Structure and function

**Fig. 2:**
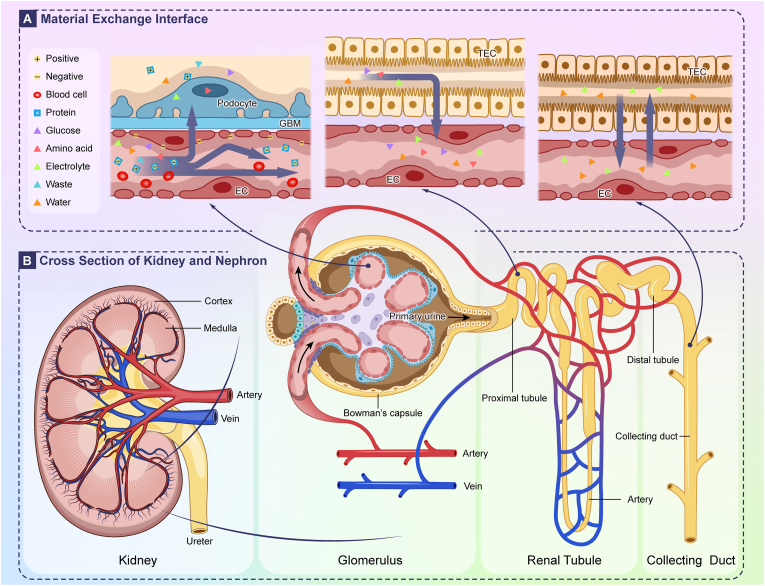
Diagram of Kidney Cross Section, Nephron Structure, and Substance Exchange Barriers in Glomerulus, Renal Tubule, and Collecting Duct. (A) Substance exchange barriers: The glomerular filtration barrier (podocytes, GBM, EC) filters blood into primary urine (blocking large molecules); the tubular reabsorption barrier (TEC, EC) reabsorbs useful substances, while the collecting duct barrier (TEC, EC) adjusts water/electrolytes to finalize urine composition. (B) Overall structure of kidney and nephron: The kidney includes cortex, medulla, plus artery (blood in), vein (blood out) and ureter (urine drain); the nephron (kidney's functional unit) consists of glomerulus (initiates primary urine with Bowman's capsule), renal tubule (aids reabsorption) and collecting duct (regulates final urine).

The functional unit of the kidney is the nephron [34,35], each consists of two main components: the renal corpuscle and the renal tubule [36]. The renal corpuscle, formed by the glomerulus enveloped within Bowman's capsule, is responsible for blood filtration [37,38]. The renal tubule, together with the collecting duct, accomplishes the reabsorption and secretion of the filtrate [[39], [40], [41]] (as illustrated in Fig. 2). The renal barrier system is comprised of three principal units, each with distinct structural and functional characteristics that directly inform OoC design goals [2,42] (summarized in Table 1).

**Table 1:** Key characteristics of renal barriers and corresponding OoC design objectives.

Renal Barrier Unit		Core Structural Features	Primary Physiological Functions	Key OoC Design Objectives
GFB	Endothelial cell layer	• Fenestrae: diameter ∼70–100 nm [43]. • Surface glycocalyx: negatively charged, covering the fenestrae.	• Primary filtration: Allows passage of water and small solutes, blocks blood cells. • Charge barrier: Repels plasma proteins via negative charge.	• Recapitulate the trilaminar interface: Achieve co-culture of endothelial cells and podocytes on opposite sides of a biomimetic porous membrane. • Simulate the dynamic mechanical environment: Inducing podocytes to form functional foot processes and slit diaphragms under mechanical stimulation.
	glomerular basement membrane (GBM)	• Nanofibrous network: Primarily composed of type IV collagen, laminin, and heparan sulfate proteoglycans. • Thickness: Native GBM is ∼330 nm thick.	• Core filtration barrier: Achieves dual selectivity based on size and charge via a molecular sieve (pore size <4 nm), preventing the leakage of large proteins (e.g., albumin).	
	Podocyte layer	• Foot processes: Cells extend secondary foot processes, interdigitating on the outer aspect of the GBM. • Slit diaphragm (SD): A ∼4–6 nm thick membrane bridging the ∼25–40 nm wide slits between adjacent foot processes; the ultimate physical barrier to filtration.	• Final regulatory filtration: The SD is the key executor of the molecular sieve function. • Dynamic support: Regulates the filtration surface area via cytoskeleton-mediated remodeling of foot processes. • Secretes extracellular matrix: Participates in GBM metabolism and maintenance.	
TRB	Proximal tubule	• Brush border: Densely packed microvilli on the luminal surface, greatly increasing the absorptive area. • Cell polarity: Abundant basolateral membrane infoldings and mitochondria for sufficient energy supply.	• Main site of reabsorption: Reabsorbs the vast majority of glucose, amino acids, proteins, water, and inorganic salts (e.g., 60–70 % of Na^+^). • Organic acid secretion: Excretes drugs (e.g., penicillin) and metabolic wastes.	• Maintain cell polarity: Culture renal tubular epithelial cells under dynamic culture conditions to maintain polarity and brush border integrity. • Apply physiological shear stress: Mimic the low level of shear stress within the tubular lumen to support transport function and mechanosensitive signaling.
	Loop of Henle	• U-shaped structure: Comprises the thick descending limb, thin limb, and thick ascending limb. • Thin-walled segment: The epithelial cells in the thin limb are flattened, facilitating water and ion exchange.	• Countercurrent multiplication: Establishes the hyperosmotic gradient in the renal medulla through selective permeability to different substances (e.g., water, NaCl), which is crucial for urine concentration.	
	Distal tubule	• Structure similar to the thick ascending limb of the loop of Henle, but with fewer basolateral infoldings and mitochondria.	• Fine-tuning of ion balance: Under the regulation of hormones like aldosterone, it continues to reabsorb Na^+^ and water, and secretes K^+^, H^+^, and NH_3_, which is essential for maintaining electrolyte and acid-base balance.	
CDRB	Cortical region	• Two epithelial cell types: Composed of principal cells (responsible for water/sodium reabsorption and potassium secretion) and intercalated cells (responsible for acid-base balance).	• Final regulation of urine: The last site for the kidney to regulate water, electrolyte, and acid-base balance. • Precise hormonal regulation: Principal cells are regulated by antidiuretic hormone (ADH) and aldosterone; intercalated cells regulate acid-base balance by secreting H^+^ or HCO_3_^−^.	• Achieve multicellular co-culture: Include both principal cells and intercalated cells in the chip model. • Introduce hormonal regulation: Integrate stimulation modules for key hormones such as ADH and aldosterone. • Establish an osmotic gradient: Create and maintain an osmotic pressure environment mimicking the medullary interstitium to study urine concentration mechanisms.
	Medullary region	• Increased epithelial cell height, predominantly consisting of principal cells.	• Urine concentration: In the hyperosmotic medullary environment, reabsorbs water under the action of ADH, ultimately completing urine concentration.	

#### 2.1.1 Glomerular filtration barrier

The GFB represents the core unit of the nephron responsible for blood filtration [5,44]. Its precise trilaminar structure is key to achieving the dual selectivity of filtration based on molecular size and charge, making it a primary focus and significant challenge for chip simulation.

The endothelial cell layer is characterized by fenestrae (∼70–100 nm) and a surface glycocalyx that acts as a charge barrier [43]. The GBM provides a structural and charge barrier rich in type IV collagen, laminin, and heparan sulfate proteoglycans [[45], [46], [47]]. The podocyte layer forms the final barrier, with interdigitating foot processes bridged by the SD, a dynamic protein sieve (e.g., nephrin, podocin) with a pore size of ∼4–6 nm [48,49].

The critical aspects for constructing an in vitro glomerular barrier model on a chip are: Reproducing endothelial fenestral structures with physiological pore size and charge characteristics in vitro and ensuring their stability under blood flow conditions; Utilizing biomimetic nanomaterials to simulate the nanofibrous meshwork, mechanical properties, thickness, and critical negative charge distribution of the GBM; Inducing or sustaining podocytes in vitro to form complex and functional foot process structures and an intact slit diaphragm, while also mimicking their dynamic responses to physiological or pathological mechanical stimuli.

#### 2.1.2 Renal tubular reabsorption barrier

The renal tubule, particularly the proximal segment, is lined by a polarized epithelial monolayer [50,51]. Proximal tubule epithelial cells (PTECs) exhibit a highly polarized structure with an apical brush border [52,53], which can maximize reabsorptive surface area and express a repertoire of specific transporters and channels (e.g., Na^+^/K^+^-ATPase, Sodium-Glucose Cotransporter 2 (SGLT2)) [[54], [55], [56], [57], [58]]. Tight junctions between cells form a selectively permeable paracellular barrier [59,60].

Constructing an in vitro renal tubular barrier requires: Inducing and maintaining the apical-basal polarity, functional brush border structure, and tight junction integrity of PTECs within a dynamic fluid environment to support their active transport and secretory functions. Simulating the low level of shear stress generated by luminal fluid flow, which is crucial for maintaining cell polarity and activating mechanosensitive signaling pathways [61].

#### 2.1.3 Collecting duct regulatory barrier

The collecting duct, the terminal segment of the nephron, receives fluid from the distal tubules of multiple nephrons [62,63]. It fine-tunes urine composition through the coordinated action of principal cells and intercalated cells [64]. Principal cells mediate water reabsorption via Aquaporin-2 (AQP2) and sodium/potassium balance, regulated by hormones like aldosterone and vasopressin (ADH) [[65], [66], [67]]. Intercalated cells (type A and B) regulate acid-base balance by secreting H^+^ or HCO_3_^−^ [68,69].

In vitro simulation of the collecting duct necessitates attention to: Its cell type specificity, requiring co-culture of principal and intercalated cells. Its ability to respond to key hormones such as aldosterone and ADH. The establishment of an osmotic pressure gradient to study the mechanisms underlying urine concentration.

### 2.2 Pathologic correlates of renal barrier dysfunction

As outlined earlier, disruption of the renal barrier's structural and functional integrity is a hallmark of numerous kidney diseases. Understanding these pathologic mechanisms provides a direct blueprint for designing disease-specific OoC models, which must be capable of introducing targeted pathological stimuli and monitoring the resultant functional decline.

For instance, Diabetic kidney disease (DKD) is driven by persistent hyperglycemia, which promoting podocyte oxidative stress, generating advanced glycation end-products (AGEs) that disrupt the GBM charge barrier and induce thickening, and impairing critical podocyte-endothelial crosstalk [40,[70], [71], [72]]. To model DKD, OoC platforms must enable sustained exposure to high glucose concentrations, allow for the assessment of GBM thickening, and quantitatively monitor the consequent increase in barrier permeability to albumin and other macromolecules.

Hereditary nephropathies like Alport syndrome is because of genetic mutations, such as those in genes encoding type IV collagen alpha chains or the slit diaphragm proteins nephrin and podocin, which directly result in structural defects in the GBM or impaired assembly/function of the SD [[73], [74], [75]]. This severely compromises molecular sieving function and often leads to significant disorder early in the disease course [76,77]. These diseases are ideally modeled using patient-specific or Clustered Regularly Interspaced Short Palindromic Repeats (CRISPR)/Cas9-genetically engineered iPSCs differentiated into target cells [78,79]. The OoC serves as a platform to phenotypically validate the functional consequences of these mutations, like altered filtration selectivity, and test potential interventions.

Ischemic or nephrotoxic insults (e.g., from cisplatin or aminoglycosides) trigger acute kidney injury (AKI) by disrupting tubular tight junctions and cellular polarity, leading to barrier dysfunction and filtrate back-leakage [[80], [81], [82], [83]]. Tubule-on-a-chip models for AKI research require the application of precise ischemic or chemical insults. Key readouts include real-time tracking of transepithelial electrical resistance (TEER) decline, tight junction protein integrity like ZO-1 localization, and the release of specific injury biomarkers like kidney injury molecule-1 (KIM-1).

The complex architecture and specialized functions of the renal barrier present a formidable challenge for in vitro modeling. Understanding these specific pathological mechanisms that disrupt key components of the renal barrier not only underscores the critical importance of accurately mimicking healthy barrier function in vitro, but also directly informs the construction strategy for renal barrier OoC models intended for disease modeling. The primary goal of renal barrier OoC technology is to reconstruct these essential properties through engineering approaches, thereby enabling reliable simulation of both physiological and pathological processes.

In summary, the renal barrier, particularly the GFB, is characterized by its precise 3D spatial configuration (endothelium-basement membrane-podocytes), synergistic action of molecular sieving and charge selectivity, dynamic substance transport functions dependent on mechanical and biochemical signals, and highly polarized epithelial cell properties. It is central to maintaining renal homeostasis. This renal barrier represents a critical challenge that has proven difficult to accurately simulate using traditional in vitro models. The overarching goal of constructing renal barrier biochips is to reconstruct these key biological properties in vitro through engineering approaches, ultimately achieving reliable simulation of physiological functions and pathological mechanisms.

## 3 Renal barrier biochip construction strategy: accurate modeling of key biological structures and functions

The core objective in constructing renal barrier models using OoC technology is to accurately recapitulate their key biological properties through a multidisciplinary integration of microengineering, biomaterials design, and cellular biology. These properties encompass the precise spatial configuration, specialized cellular functions, and the dynamic physicochemical microenvironment of the renal barrier [84,85]. Such features are exemplified by the glomerulus's trilaminar interface (spatial configuration), the formation of the podocyte slit diaphragm and tubular epithelial polarity (cellular functions), and blood flow-induced shear stress, transmembrane pressure, and biochemical gradients(physicochemical microenvironment) [86,87]. Achieving this goal necessitates the systematic integration of multidisciplinary methodologies spanning materials engineering, micromachining technology, cell biology, and microfluidic control. The construction strategy revolves around three interconnected dimensions: selecting and processing appropriate biocompatible materials to form biomimetic substrates and structures; designing microstructures capable of reproducing the spatial configuration and interfaces of the renal barrier, coupled with the selection and introduction of functional cellular models; and ultimately, implementing the dynamic simulation of the physiopathological microenvironment.

### 3.1 Material and manufacturing process

#### 3.1.1 Material chemistry and surface functionalization

The selection of chip materials and the employed manufacturing processes directly influence biocompatibility, structural biomimicry, and functional reliability. Ideal materials must balance optical transparency for real-time observation, mechanical properties to simulate basement membrane stiffness or vascular elasticity, surface chemistry affecting cell adhesion and function, long-term stability, and processing feasibility. Currently, polymeric materials and hydrogels dominate due to their favorable overall performance and processing flexibility. A critical consideration in material selection is how their intrinsic properties—such as stiffness, permeability, and drug-binding capacity—align with the physiological parameters of native renal barriers to ensure accurate functional emulation (as compared in Table 2).

**Table 2:** Comparative Physicochemical and Mechanical Properties of OoC Materials vs. Native Human Renal Tissues.

Property Category	Parameter	Native Human Renal Tissue	PDMS	PMMA	PC	PET	Natural Hydrogels (Collagen/Gelatin/Silk Fibroin)	Synthetic Hydrogel (PEGDA)
**Mechanical Characteristics**	**Mechanical Stiffness**	- Glomerulus: ∼2.5 kPa - Proximal Tubule: ∼1–3 kPa - Renal Interstitium: ∼5–10 kPa	Soft, tunable; ∼1–10 MPa (flexible, mimics vascular pulsation)	Rigid; ∼2–3 GPa (stable for long-term culture)	Rigid; ∼2.4 GPa (high mechanical stability)	Rigid; ∼2.7 GPa (excellent tensile strength)	Tunable; ∼0.5–10 kPa (matches glomerular/tubular stiffness)	Tunable; ∼1–20 kPa (via photocrosslinking density)
	**Elastic Modulus**	- Glomerulus: ∼2–3 kPa - Tubules: ∼1–2 kPa	∼0.3–1.5 MPa (elastic, supports cyclic strain)	∼2.5–3.5 GPa (brittle, low deformability)	∼2.2–2.6 GPa (moderate elasticity for membrane fabrication)	∼2.5–3.0 GPa (high elastic recovery)	∼0.1–10 kPa (collagen: ∼0.1–1 kPa; silk fibroin: ∼2–8 kPa)	∼1–15 kPa (photocrosslinking-dependent)
**Transport Properties**	**Permeability (Small Molecules/Water)**	- Glomerulus: High (filters water/small solutes, retains albumin) - Tubules: Selective (reabsorbs glucose/ions)	High (gas/water permeable; supports cell respiration)	Low (impermeable to small molecules; requires porous membranes)	Moderate (porous membranes: 0.4–8 μm pores; supports filtration)	Moderate (porous membranes: 0.2–10 μm pores; good solute exchange)	High (hydrophilic network; supports nutrient/waste diffusion)	Moderate (tunable via crosslinking; controlled solute transport)
	**Permeability (Macromolecules/Proteins)**	- Glomerulus: Low (retains albumin >99 %) - Tubules: Low (tight junctions block proteins)	Low (hydrophobic surface may trap proteins; reduced via PEG coating)	Very Low (chemically inert; minimal protein adsorption)	Low (smooth surface; low protein binding)	Low (surface modifiable with ECM to reduce protein adsorption)	Low (biocompatible network; minimal non-specific protein binding)	Low (functionalizable with ligands to control protein interaction)
**Optical Characteristics**	**Optical Transparency**		Excellent (>90 % transmittance; enables real-time imaging)	Excellent (>92 % transmittance; suitable for high-resolution microscopy)	Good (∼88 % transmittance; compatible with fluorescence imaging)	Good (∼85 % transmittance; stable under long-term light exposure)	Good (∼70–90 % transmittance; depends on crosslinking density)	Good (∼80–95 % transmittance; photopolymerizable for clear structures)
**Biological Compatibility**	**Cell Adhesion**		Poor (hydrophobic; requires oxygen plasma/ECM coating)	Moderate (requires ECM coating for renal cell adhesion)	Moderate (porous membranes support cell seeding; needs ECM modification)	Good (surface coatable with collagen/laminin; supports epithelial polarization)	Excellent (mimics native ECM; supports podocyte foot process formation/tubular polarity)	Moderate (functionalizable with RGD peptides to enhance cell adhesion)
	**Cytotoxicity**		Low (leaches uncured oligomers; reduced via baking)	None (chemically stable; no leachables)	None (biocompatible; used for porous cell culture inserts)	None (stable; non-toxic for long-term cell culture)	None (biodegradable; low inflammatory response)	None (photopolymerizable with low-toxicity initiators)
**Chemical Stability**	**Drug Adsorption**		High (hydrophobic; adsorbs hydrophobic drugs/metabolites)	Low (chemically inert; minimal drug binding)	Low (resistant to drug adsorption; suitable for toxicity testing)	Low (stable surface; low drug retention)	Low (hydrophilic; minimal non-specific drug binding)	Low (controlled chemistry; no drug adsorption)
	**Degradability**		Non-degradable (stable for >6 months in culture)	Non-degradable (resistant to enzymatic/hydrolytic degradation)	Non-degradable (stable under physiological conditions)	Non-degradable (high resistance to degradation)	Controlled (collagen: ∼2–4 weeks; silk fibroin: ∼1–3 months)	Tunable (slow degradation; ∼1–6 months via crosslinking)

Polydimethylsiloxane (PDMS) has become the material of choice for building flexible kidney biochip structures, particularly for mimicking vascular pulsation or dynamic responses of the basement membrane, owing to its high elasticity, excellent optical transparency, and well-established soft lithography processing [[88], [89], [90], [91]]. Through soft lithography, molds featuring complex microchannels, chambers, and porous membranes can be rapidly replicated in PDMS [92]. These structures are essential for separating different cell types and simulating filtration interfaces [93,94]. However, the inherent hydrophobicity of PDMS tends to adsorb hydrophobic drug molecules or metabolites, potentially compromising the accuracy of drug testing [95]. Consequently, surface modification techniques, such as oxygen plasma treatment or hydrophilic coatings like polyethylene glycol (PEG), are typically required to mitigate this limitation [89,96,97]. Most contemporary renal barrier biochips utilize PDMS as the primary flexible substrate material [[98], [99], [100], [101]]. Some researchers have further fabricated PDMS into flexible porous membranes to validate filtration barrier function by facilitating dynamic interactions between endothelial cells and podocytes [102,103].

For applications demanding greater mechanical stability, chemical resistance, or long-term incubation, other polymer materials demonstrate unique value. Poly(methyl methacrylate) (PMMA) and polycarbonate (PC) are prized for their excellent optical properties, chemical stability, and precision machining characteristics. These two polymers are commonly used to fabricate rigid chips or serve as porous membrane substrates, especially in scenarios requiring high-resolution imaging or reusability [[104], [105], [106]]. PC membranes, with their uniform and controllable pore sizes typically ranging from 0.4 to 8 μm and good oxygen permeability, are frequently selected to mimic porous interfaces for filtration or separation barriers [107,108]. Polyethylene terephthalate (PET) is known for its excellent mechanical stability and surface modifiability, such as ease of coating with extracellular matrix (ECM) proteins. It provides a reliable substrate for constructing long-term chronic kidney disease models and is often employed to fabricate porous membranes that separate epithelial and endothelial cells [[109], [110], [111]]. Cycloolefin polymers (COP) also find application, particularly in monitoring contexts, due to their optical transparency [107,112].

Hydrogel materials, especially natural-origin ones like collagen, gelatin, and silk fibroin, are gaining increasing importance in constructing 3D tissue structures that more closely resemble the in vivo cellular microenvironment [[113], [114], [115], [116]]. This stems from their excellent biocompatibility, tunable mechanical properties capable of mimicking different tissue stiffnesses, and the ability to encapsulate cells [[117], [118], [119]]. For instance, stiffness-tunable gelatin-based hydrogels have been successfully used to demonstrate that podocytes differentiate optimally on substrates matching the physiological stiffness of the GBM (∼2–5 kPa) [120]. Silk fibroin, characterized by high strength, controlled degradability, and the capacity to form ultrathin porous structures, represents an ideal candidate for mimicking basement membranes [[121], [122], [123]]. Synthetic hydrogels like poly(ethylene glycol) diacrylate (PEGDA), conversely, can be photopolymerized, enabling the printing of high-resolution complex structures and facilitating easy functionalization through modification [[124], [125], [126]].

Beyond bulk material properties, the surface chemistry of chip substrates plays a decisive role in directing cell behavior. Introducing specific chemical groups (e.g., -NH_2_, -SH, catechols) or bioactive motifs (e.g., Arg-Gly-Asp (RGPMMAD) peptide) via surface functionalization significantly enhances cell adhesion, focal adhesion formation, and subsequent proliferation [126,127]—critical for establishing stable and functional renal barriers.

**Fig. 3:**
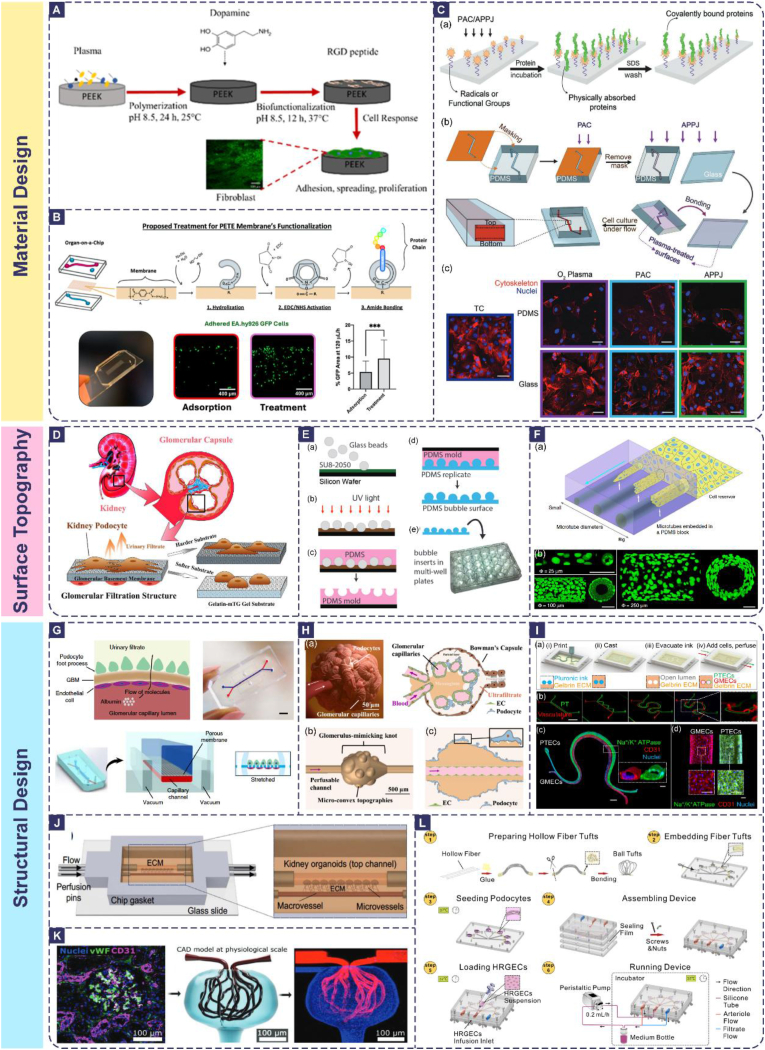
Representative Renal Barrier Chip Designs. (A) RGD peptide functionalization of PEEK surfaces via a polydopamine coating improves biocompatibility and cell response [131]. (B) Functionalization of PETE membranes for the enhancement of cellular adhesion in Organ-on-a-Chip devices [132]. (C) A novel microfluidic platform that combines two plasma surface treatments: PAC and APPJ, enable reagent-free covalent immobilization of biomolecules is described here [138]. (D) Stiffness-tunable gelatin-mTG hydrogel provides an ideal platform to study kidney podocyte mechanotransduction [120]. (E) the modified photolithography and micromolding process used to prepare the micro-hemispherical “bubble” topography for podocyte cultivation [193]. (F) MDCK cells are seeded on a fibronectin reservoir in front of a PDMS block containing cylindrical microtubes of different sizes. The cells start crawling into the openings of the microtubes once they are in full confluenc [195]. (G) Design of microfluidic Organ Chip device to recapitulate the structure and function of the kidney glomerular capillary wall [103]. (H) Design of the biologically inspired microfluidic extruded topographic hollow fiber (h-FIBER), consisting of a vessel-like perfusable tubular channel and a glomerulus-like knot with microconvex topography on its surface [199]. (I) Fabrication of 3D VasPT Models via Sacrificial Printing [156]. (J) Formation of channels in the chip via pre-placed inner pins, which are removed after matrix solidification [151]. (K) Multiphoton-guided creation of 3D cellularized microvessels [165]. (L) Schematic of the construction steps of the glomerulus chip, where bundles of hollow fibers were spherically twisted and embedded in designed Bowman's capsules to form spherical glomerular capillary tufts [108].

These modifications work by providing high-affinity ligands for cell surface integrin receptors. The binding of ligands like the Arg-Gly-Asp (RGD) peptide to integrins initiates intracellular signaling cascades, primarily through focal adhesion kinase (FAK), which promotes the assembly of mature focal adhesions and the subsequent recruitment of actin cytoskeletal proteins [128,129]. This strong integrin-mediated adhesion not only ensures stable cell anchorage but also transmits pro-survival and pro-proliferative signals, fundamentally enhancing cell viability and function beyond what is possible on unmodified, bioinert surfaces. For instance, Salsano et al. demonstrated that coating 3D-printed PCL scaffolds with an RGD-functionalized polymer derivative significantly enhanced SAOS-2 cell adhesion and mineralization compared to controls, a direct result of this specific ligand-integrin engagement and the resultant activation of downstream mechanotransduction pathways [130]. Similarly, Tapia-Lopez et al. showed that RGD peptide functionalization of PEEK surfaces via a polydopamine coating progressively improved fibroblast attachment, spreading, and proliferation with each modification step, correlating with enhanced integrin clustering and focal adhesion complex formation (Fig. 3A) [131]. Likewise, covalent immobilization of proteins onto chemically activated surfaces, like EDC/NHS chemistry on hydrolyzed PETE) membranes as in Sobejano de la Merced et al. (Fig. 3B) [132], provides a stable and robust interface, which significantly outperforms simple protein adsorption [133]. This approach effectively supports endothelial and epithelial cell adhesion under flow by preventing the reversible detachment of adsorbed proteins and ensuring sustained presentation of native cell-adhesive motifs [134,135].

Beyond wet-chemical methods, advanced physical methods such as plasma surface treatment offer a reagent-free alternative for covalent biomolecule immobilization [136,137]. Ashok et al. developed a novel microfluidic platform combining Plasma-Activated Coating (PAC) and an Atmospheric Pressure Plasma Jet (APPJ), which enabled reagent-free covalent immobilization of fibronectin on PDMS and glass(Fig. 3C) [138]. This approach, surpassing the instability of O_2_ plasma treatment, significantly improved endothelial cell attachment and proliferation by creating a stable, covalently bound bioactive layer that robustly engages with cell integrins.

These modifications provide specific ligands for integrin receptors, initiating the assembly of focal adhesion complexes and activating downstream signaling pathways that are essential for establishing stable and functional renal barriers.

#### 3.1.2 Manufacturing process

The choice of fabrication technique depends on the complexity and precision requirements of the target structure. Soft lithography, involving casting and molding using SU-8 photoresist masters, efficiently produces chip bodies containing microchannels, chambers, and porous membranes [[139], [140], [141]]. It remains the mainstream process for constructing OoCs incorporating porous membranes and complex flow paths [100,142,143]. However, its resolution is limited compared to advanced techniques, making it challenging to replicate the sub-micron features of the glomerular basement membrane or the intricate 3D architecture of the nephron.

This limitation is being overcome by a suite of advanced manufacturing technologies that represent key breakthroughs for renal barrier models. 3D printing technology, particularly extrusion-based and vat photopolymerization-based bioprinting, overcomes the geometric constraints of traditional methods [[144], [145], [146]]. Leveraging different bio-ink materials, multiple printing processes, and flexible 3D spatial design [147], it enables the direct fabrication of biomimetic structures with complex 3D morphologies, such as convoluted tubules or branching vascular networks [[148], [149], [150]]. This capability provides a powerful tool for constructing renal barrier models that are highly vascularized and spatially organized closer to the physiological state [[151], [152], [153]]. For instance, sacrificial inks can be printed to create lumen templates, which, after embedding in hydrogel and subsequent dissolution, allow cell seeding to form open tubular structures [150,154]. This approach has been used to create convoluted proximal tubules with enhanced physiological functionality compared to 2D models [155,156]. Homan et al. utilized sacrificial ink to print convoluted tubule lumens with a curvature radius of approximately 50 μm. This approach yielded proximal tubule cell apical membrane microvilli densities closer to in vivo level, and the epithelial morphology and functional characteristics are significantly enhanced [157].

Alternatively, Multiphoton Laser Micromachining (or ablation) offers a paradigm shift in achieving submicron precision and unparalleled design freedom [[158], [159], [160]]. This technique transcends the layer-by-layer approach of most printing, allowing for the direct, maskless writing of complex 3D microarchitectures within hydrogels [161,162]. Its supreme utility is demonstrated in the fabrication of complex, perfusable, organ-specific capillary networks that approach the scale and branching complexity of native renal vasculature, which remains a formidable challenge for soft lithography [163,164]. Rayner et al. successfully employed this method to create and perfuse cellularized human kidney microvascular beds, showcasing its potential for building high-fidelity vascularized models(Fig. 3K) [165]. For hard materials like silicon, glass, or PMMA, photolithography and etching remain key methods for realizing high-precision sub-micron structures, such as ultrathin films or nanopores, albeit at higher cost and often requiring cleanroom environments [[166], [167], [168]].

The ultimate goal of materials and fabrication processes is to create physical platforms that can stably support cell growth, accurately reproduce barrier spatial configurations, particularly critical biomimetic porous membranes or interfaces, and allow for the integration of dynamic control modules. This lays the essential groundwork for subsequent cell culture and microenvironment simulation.

### 3.2 Cell type and origin

The selection and integration of appropriate cell types onto the defined biomimetic structures are critical for realizing barrier function. The choice of cell source requires careful consideration of physiological relevance, accessibility, expansibility, and long-term stability.

Podocytes, as the core of the GFB, present the greatest challenge, specifically the formation of functional foot processes and an intact slit diaphragm [169,170]. Their limited proliferative capacity and high degree of specialization often constrain glomerular modeling [2]. Obtaining physiologically relevant podocytes through reliable methods is crucial to accelerate the related research. Currently, primary podocytes isolated directly from human kidney tissues offer high physiological relevance but are difficult to obtain, and primary cells are challenging to maintain long-term in vitro with preserved functions [26,171]. Immortalized cell lines are easier to culture and possess indefinite proliferative capacity, but they are often functionally limited, with expression levels of key genes typically lower than in primary cells [2]. Therefore, directed differentiation of pluripotent stem cells, including iPSCs or embryonic stem cells (ESCs), represents another vital approach for obtaining podocytes [172]. This strategy addresses sourcing issues and enables patient specificity, achieving differentiation efficiencies exceeding 90 % [102]. However, achieving their full differentiation towards a mature phenotype requires further investigation. Given the challenges in sourcing and maintaining functional podocytes, exploring reliable methods remains a priority.

Glomerular endothelial cells (GEnCs) must possess fenestral structures and participate in charge selectivity [173]. PTECs require well-established apical-basal polarity and transporter function [52]. Similar to podocytes, these cells also originate from three main sources: primary cells, immortalized cell lines, and iPSC/ESC-differentiated cells [174].

Vascular endothelial cells, used to construct vascular networks within renal biochips and typically simulating the blood-tissue interface, often utilize models like human umbilical vein endothelial cells (HUVECs) isolated from umbilical veins [175,176].

Some researchers have explored co-culturing cells of multiple origins. For example, co-culturing proximal tubule cells derived from human iPSC-based kidney organoids with immortalized proximal tubule cells resulted in models exhibiting higher transporter protein expression levels, enhanced reabsorption and efflux capabilities, and tighter intercellular junctions [110,177]. Furthermore, iPSC technology enables the differentiation of multiple renal cell types, such as podocytes, endothelial cells, and tubular epithelium, from the same donor. This provides a revolutionary pathway to construct genetically background-consistent, patient-specific models of intact nephrons, greatly facilitating personalized disease modeling and drug response studies [178]. Nevertheless, achieving the full maturity (e.g., complex foot process structures in podocytes) and ensuring the long-term functional stability of iPSC-differentiated cells remain key research foci requiring continuous improvement within the field [179].

Beyond traditional 2D monocultures or co-cultures, spheroids, organoids, and 3D culture systems serve as critical intermediate or complementary models in renal barrier OoC research, bridging the gap between static cell cultures and fully integrated chip systems [180,181]. Spheroids are self-assembled 3D aggregates of single or multiple renal cell types, characterized by their ability to recapitulate local cell-cell interactions and rudimentary ECM microenvironments [182]. For instance, 3D acini formed by self-aggregation of MDCK-2 cells exhibit significant increases in the average tension per molecule for each protein compared to 2D monolayers [183].

Kidney organoids, particularly those derived from iPSCs or ESCs, represent a more complex 3D culture system that recapitulates the multi-cellular composition and structural organization of the native nephron. These organoids self-organize into glomerulus-like structures (with podocytes, GEnCs, and mesangial cells), segmented tubules (proximal, distal, and collecting duct-like), and interstitial cells, closely mimicking the spatial architecture of the renal cortex [184]. Their key advantage lies in providing a "miniature renal tissue" that retains patient-specific genetic backgrounds [185]. When integrated into OoC platforms, these organoids can be further matured by chip-specific dynamic stimuli: perfusion with physiological shear stress or cyclic strain significantly enhances organoid vascularization and upregulates mature markers [186].

### 3.3 Structural design and microenvironment simulation strategies

The core advantage of OoC technology over traditional static models lies in its ability to accurately simulate the dynamic physical and chemical microenvironment to which the renal barrier is exposed in vivo. This dynamic simulation is fundamental to conferring physiological relevance to the microchip model and achieving high-fidelity functional reproduction.

#### 3.3.1 Physical-mechanical microenvironment simulation

In addition to material and cell cues, simulating the physical-mechanical microenvironment focuses on recreating the key signals experienced by renal tissues.

The physical topography of the substrate is another powerful cue to guide cell migration and tissue organization, working in concert with biochemical signals. Cells sense these physical features through a process called contact guidance, where anisotropic topographies direct cytoskeletal alignment and focal adhesion distribution, leading to polarized cell morphology and migration.

**Matrix Stiffness Mimicry:** Matrix stiffness is a critical physical signal regulating kidney cell proliferation, migration, survival, and differentiation. Under physiological conditions, glomerular stiffness is approximately 2.5 kPa, which decreases in various kidney diseases but can increase in pathologies like diabetic nephropathy due to fibrosis and glycation [[187], [188], [189]]. To mimic this property, researchers employ tunable stiffness hydrogels, such as polyacrylamide (PAA) and gelatin modified with microbial transglutaminase (gelatin-mTG), to construct chip substrates. Elastic modulus is controlled via methods like photocrosslinking [190]. Demonstrating this, Hu et al. used a gelatin-mTG platform to show that podocytes differentiated optimally on substrates with intermediate stiffness (2–5 kPa), exhibiting a 3-fold increase in Podocin expression compared to soft or hard substrates. Conversely, high stiffness (>10 kPa) induced podocyte cytoskeletal reorganization, mimicking early lesion characteristics of diabetic nephropathy(Fig. 3D) [120].

**Surface Topography Design:** The surface topography and curvature of the basement membrane significantly influence cell polar arrangement. Curved surfaces mimicking the in vivo microenvironment can be created within biochips using techniques like template replication or 3D printing [191,192]. Korolj et al. prepared molds by embedding glass microbeads less than 100 μm in diameter into silicon wafers. The resulting imprinted PDMS possessed porous curved surface properties. After podocyte seeding, this design led to a 40 % increase in the density of foot process extensions, up-regulation of the Nephrin gene, and significantly promoted slit diaphragm formation(Fig. 3E) [193]. Similarly, Bosch-Fortea et al. demonstrated that MDCK cells confined on narrow (15 μm wide) micropatterns formed tall, narrow lumens, highlighting how geometric constraints directly dictate tubular morphology and epithelial architecture [194].

**Restricted Geometric Space Modulation:** Geometric constraints influence renal tubule cell structure and behavior by modulating cell spreading area. Micropatterning techniques like photolithography or soft lithography allow the construction of microchannels different wide within the biochip to mimic the physical constraints of the tubular lumen [51]. Xi et al. simulated the migration of renal tubular epithelial cells within microtubules with diameters equivalent to 1–10 cell lengths. This setup recapitulated physiological apical-basal polarity, with cell actin alignment, orientation, organization, and migration patterns dependent on the degree of tubular constraint or bending(Fig. 3F) [195].

**Fluid Shear Simulation:** Blood flow-induced shear stress is a core dynamic stimulus maintaining renal barrier function, and microfluidics provides the basis for generating precise, continuous flow fields. Glomerular endothelium requires relatively high shear stress (0.1–0.2 dyn/cm^2^) to maintain fenestral structure, whereas the proximal tubule requires only low shear stress, around 0.2 dyn/cm^2^, to activate ciliary mechanotransduction [196,197]. The tapered microchannel design, with a width gradient (inlet 1000 μm → outlet 300 μm), mimics the difference in the degree of contraction between afferent and efferent glomerular arterioles. By adjusting the perfusion flow rates (5, 10, and 15 μL/min), Zhou et al. established shear stresses of 0.001, 0.002, and 0.003 dyn/cm^2^ in the channel utilizing this structure. This experimental setup recapitulated the stress effects of glomerular capillaries in hypertensive nephropathy in vitro and successfully simulated the pathology of hypertension-induced proteinuria [198]. Furthermore, Xie and colleagues developed a topographical hollow fiber (h-FIBER) with glomerulus-mimicking knot-like structures using microfluidic spinning technology, which overcomes the geometric limitations of planar chips. The h-FIBER comprises a perfusable tubular channel (for endothelial cell cultivation to mimic the vascular lumen) and a glomerulus-like knot with microconvex topography on its surface (for podocyte cultivation). Compared with planar models, podocytes on the h-FIBER can exert better barrier function, achieving geometric biomimetic enhancement of molecular sieve function(Fig. 3H) [199].

**Transmembrane Pressure and Cyclic Strain Simulation:** Pressure changes during glomerular filtration also impact podocytes, with hypertension increasing mechanical stress leading to podocyte loss [[200], [201], [202]]. Chen et al. designed a dual-chamber biochip where the 500 kDa dextran filtration was blocked at 30 mmHg. When the transmembrane pressure reached 60 mmHg, 500 kDa dextran began to penetrate the filtration membrane due to the disorganized actin cytoskeleton and downregulated synaptopodin in podocytes, mimicking hypertension-induced glomerular leakage [203]. In more complex models, certain designs have further incorporated periodic mechanical strain or pneumatically driven pulsatile flow. Through multimechanical coupled chambers, they have addressed the challenge of synergistic simulation of pulsatile blood flow and transmembrane pressure, aiming to simulate the impact of pulsatile pressure generated by cardiac pulsation on renal blood flow and filtration [204]. Musah et al. designed a dual-chamber chip where the upper urinary channel is subjected to a shear stress of 0.0007 dyn/cm^2^, while the lower vascular channel is subjected to a shear stress of 0.017 dyn/cm^2^ to mimic blood flow. Simultaneously, 10 % cyclic strain is applied via the pneumatic membranes on both sides of the lower channel to simulate vascular pulsation [102]. This combined mechanical loading increased the maturation level of podocytes differentiated from iPSCs, as evidenced by more developed foot processes and enhanced expression of specific markers. Moreover, the chip can mimic adriamycin-induced leakage toxicity, providing a multi-parameter mechanical platform for studies on drug-induced nephrotoxicity(Fig. 3G).

#### 3.3.2 Structural design

Building upon the aforementioned physical-mechanical simulation tools, the structural design of the chip primarily focuses on designing the spatial segregation and geometric morphology of the simulated in vivo tissues. This provides the correct topological cues and interaction platforms for the cells.

**Multilayer Membrane Interface:** The most widely adopted design utilizes a flexible porous membrane, often made of PDMS, PC, or PET, coated with extracellular matrix proteins like laminin and type IV collagen [205,206]. These membrane physically separates two microfluidic channel chambers: one side is seeded with GEnCs to mimic the capillary lumen, while the other side is seeded with podocytes to mimic Bowman's space [[207], [208], [209]]. This design directly replicates the in vivo trilaminar spatial configuration (endothelium-basement membrane-podocytes) of the glomerular filtration barrier. Careful design of the membrane pore size, typically ranging from 0.4 to 7 μm, is crucial for controlling molecular filtration selectivity [3,98,110]. Optimization of membrane surface coatings, such as type IV collagen/heparan sulfate complexes, can further enhance the biomimetic properties of the basement membrane [98,103,109].

**Tubular Structures:** Simulating renal tubules necessitates creating 3D tubular structures with open lumens. Primary methods include: Preformed lumens using polycarbonate membrane-wrapped hollow fibers or pre-positioned cylindrical channels within the chip; the inner walls are coated with a matrix like collagen before seeding with PTECs(Fig. 3J) [108,151,210]. 3D bioprinting employs sacrificial materials, such as gelatin, printed into the desired shape template. This template is embedded within a hydrogel, such as gelatin-fibrinogen, and subsequently dissolved to remove the sacrificial material, forming a hollow tubular lumen [211]. PTECs are then seeded onto the inner lumen wall. This method allows more precise mimicry of natural tubule geometry(Fig. 3I) [157]. Decellularized scaffolds utilize the natural ECM from decellularized renal tissues. The preserved luminal structure and inherent biochemical signals, such as heparan sulfate, effectively guide the polar alignment and directional transporter protein expression of PTECs [32]. Some studies have attempted to construct interwoven vascular networks and renal tubular structures simultaneously within hydrogel matrices, providing initial models of the spatial relationships and interactions between blood vessels and tubules in the nephron [212]. Cutting-edge research is dedicated to constructing 3D vascular network structures approaching the complexity of real organs. Using high-precision 3D printing techniques like multiphoton laser ablation, micrometer-scale branching channel networks can be fabricated within hydrogels. Perfusing these with endothelial cells forms perfusable blood vessels, with pore sizes potentially designed within the physiological range (μm–mm) [165,[213], [214], [215]].

#### 3.3.3 Biochemical microenvironment simulation

Simulation of the biological and chemical microenvironment focuses on establishing and maintaining critical concentration gradients and gas tensions within the chip. This mimics the drivers of substance transport and the microenvironment for cellular metabolism [216]. Renal barrier function depends on precise local biochemical gradients—such as ion concentrations, metabolic wastes, hormones, and oxygen tension—for filtration, reabsorption, secretion, and regulation [23,217]. Disturbances in these gradients under pathological conditions, like hyperglycemia in diabetic nephropathy or hypoxia in ischemia-reperfusion, are direct causes of barrier dysfunction [218].

Constructing complex microfluidic pathways, such as dendritic networks, serpentine channels, or utilizing diffusion at parallel laminar flow interfaces, enables the dynamic establishment and maintenance of the desired chemical gradients within the biochip. For instance, establishing a concentration gradient of urea, creatinine, or a drug like cisplatin between the lumen and the interstitial compartment of a tubular chip can directly model the physiological processes of solute reabsorption or waste/drug secretion by tubular epithelial cells, alongside their alterations in response to toxic injury [219]. In a biochip model simulating diabetic kidney disease, Wang et al. generated a tri-channel microfluidic system to establish high-glucose conditions (5 mmol/L and 30 mmol/L). Continuous exposure of cells to these conditions induced key pathological phenotypes—increased barrier permeability, disruption of tight junctions, and podocyte migration—effectively recapitulating early structural lesions characteristic of human DKD [100].

Simulating oxygen tension gradients is essential for studying functional differences between renal regions. A significant physiological oxygen gradient exists between the renal cortex (pO_2_ ∼40–50 mmHg) and medulla (pO_2_ ∼10–20 mmHg) [220,221]. Ischemia-reperfusion injury (IRI), a primary cause of AKI, fundamentally involves the sudden interruption and restoration of oxygen supply [222,223]. Leveraging the inherent gas permeability of biochip materials, or integrating gas exchange membranes/channels in specific regions, allows precise modulation of local oxygen concentration within the biochip. For example, in a biochip model simulating IRI, Salih et al. cultured primary human renal proximal tubular epithelial cells and primary human endothelial cells on both sides of a porous membrane, respectively. They established the model using hypoxic and normoxic culture media, validated multiple RHR-specific hypoxic biomarkers, and the tests revealed that combinational vitamin therapy could reduce the incidence of RHR injury [224].

Building upon the successful simulation of the physical and chemical microenvironment, achieving high-fidelity reconstruction of renal barrier function necessitates the deliberate integration of complex biological signalling. Intercellular communication is central to maintaining barrier homeostasis [225]. For instance, the stability of the podocyte slit diaphragm relies not only on the expression of nephrin but crucially on its trans-interaction between adjacent foot processes [226,227]. This mechanism has been validated in chip models; for example, Petrosyan et al. demonstrated in a glomerulus-on-a-chip that autoantibodies from patient serum disrupt this trans-interaction, leading to proteinuria [16]. Furthermore, in models of immune-mediated injury, the introduction of patient-derived immune cells recapitulates their interaction with barrier cells via adhesion molecules like ICAM-1/VCAM-1, thereby modeling the pathogenesis of nephritis or AKI [228,229].

Besides, ECM composition and dynamic remodeling provide indispensable biochemical and structural instructions for cells. The specific GBM network of collagen IV α3/α4/α5, laminin-521, and agrin constitutes a specialized microenvironment that guides podocyte and endothelial cell polarity, differentiation, and survival [230,231]. Research shows that using substrates functionally closer to the native GBM significantly enhances model performance. For instance, Mou et al. utilized an ultrathin silk fibroin membrane, with its superior mechanical properties and biocompatibility, to successfully induce hiPSC-derived podocytes to form more mature foot processes and slit diaphragms, while also promoting the formation of fenestral structures in endothelial cells [98]. Similarly, Rayner et al. constructed a vascularized tubule unit, which employed a cell-remodelable collagen membrane that matured into a physiological exchange interface approximately 1 μm thick, composed of native basement membrane proteins and significantly enhancing glucose and albumin reabsorption [55].

The aforementioned biomimetic strategies have given rise to a plethora of sophisticated kidney-on-a-chip models with advanced functionalities. To provide a concise overview of these developments, Table 3 provides a comparative overview of the key characteristics, cellular models, functional validations, and primary applications of selected pivotal studies in the field. The pursuit of replicating the renal quintessential functions in vitro has driven several technological breakthroughs. Unlike many other organ chips where barrier integrity is the primary focus, glomerulus-on-a-chip models have pioneered the engineering of functional, permselective filters. This is exemplified by the development of biomimetic ultrathin membranes (e.g., silk fibroin [98]) and advanced interfaces like the topographical hollow fiber (h-FIBER [199]), which are specifically designed to recapitulate the in vivo molecular sieving and charge selectivity, a challenge less central to chips of barrier organs like the lung or gut. Furthermore, to model renal reabsorption and secretion, tubule-on-a-chip systems have led the way in creating perfusable, 3D convoluted tubules with open lumens using 3D bioprinting of sacrificial inks. This represents a significant geometric advancement beyond the simpler, often straight, epithelial channels common in other tissue models.

**Table 3:** Summary of Key Studies on Renal barrier Emulation using Organ-on-a-Chip Technology.

	Core Contribution/Title	Key Chip Design & Fabrication Features	Cellular Model	Key Functional Validations	Primary Application Context	Ref.
Glomerulus	Achieved flow-enhanced vascularization and maturation of kidney organoids.	3D-bioprinted gelatin-fibrin matrix; open lumens for perfusion.	hPSC-derived kidney organoids + HUVECs + Human neonatal dermal fibroblasts (HNDFs)	Increased vascular area (5x) and junction density (10x); enhanced podocyte and tubular maturation.	Nephrogenesis studies; vascularized disease modeling; regenerative medicine.	[186]
	Established a highly efficient protocol to differentiate hiPSCs into mature podocytes and integrated them into a functional glomerulus chip.	Dual-channel PDMS device with a porous PDMS membrane (50 μm thick); integrated cyclic mechanical strain (10 %).	hiPSC-derived podocytes + Human glomerular microvascular endothelial cells (GMECs)	>90 % differentiation efficiency; albumin retention >99 %, inulin filtration ∼5 %; modeled adriamycin-induced toxicity.	Nephrotoxicity screening; personalized medicine; mechanistic studies of glomerulopathies.	[102]
	Developed a tri-channel chip to model the human GFB long-term for disease modeling.	Mimetas OrganoPlate™; collagen I-filled middle channel.	Human primary/immortalized podocytes + Human glomerular endothelial cells (hGECs)	Sustained cell morphology and marker expression; serum from patients induced proportional albumin leakage.	Membranous nephropathy (MN) modeling; diabetic nephropathy studies; antibody-mediated injury.	[16]
	Employed an ultrathin Silk fibroin (SF) membrane to mediate tissue-specific morphogenesis and high-fidelity barrier function.	SF ultrathin membrane (3.5 μm); dual-channel PDMS device.	hiPSC-derived podocytes + Vascular endothelial cells	Selective molecular filtration (7 % inulin, 99.5 % albumin retention); induced fenestration-like structures in endothelium.	Drug toxicity modeling (e.g., adriamycin); basement membrane biology; high-fidelity filtration screening.	[98]
	Pioneered a spherical twisted hollow-fiber design to mimic the glomerular capillary tuft and its response to hormonal regulation.	Spherically twisted polyethylene hollow fiber embedded in a PMMA Bowman's capsule-like structure.	Human podocyte cell line + Human renal glomerular endothelial cells (HRGECs)	Demonstrated filtration regulation in response to ANP hormone; assessed cisplatin-induced nephrotoxicity.	Glomerular physiology studies; drug nephrotoxicity testing; biomimetic design.	[108]
	Reconstructed intact glomerular microtissues on-chip to model early pathological changes of diabetic nephropathy.	PDMS tri-channel device; Matrigel as GBM analog; crescent-shaped microstructures to trap glomeruli.	Rat primary glomerular microtissues (containing GECs and podocytes)	Hyperglycemia induced increased GFB permeability, elevated reactive oxygen species (ROS), and podocyte detachment.	Mechanistic studies of early DKD; drug screening for DKD.	[100]
	Modeled mechanical force-induced injury in hypertensive nephropathy via biomimetic channel design creating a pressure gradient.	Three-layer PDMS device; polycarbonate membrane; differential inlet/outlet widths to generate trans-membrane pressure.	Mouse GEnCs + Mouse podocytes (MPC-5)	Elevated pressure induced cytoskeletal rearrangement, downregulation of junction proteins, and increased macromolecule leakage.	Hypertensive nephropathy mechanisms; mechanobiology; drug testing.	[198]
	Fabricated a biomimetic GFB using an electrospun nanofibrous membrane with high structural and functional fidelity.	Poly-L-lactic acid (PLLA) electrospun nanofibrous membrane (∼7 μm); polydopamine-gelatin surface functionalization; 3D-printed bioreactor.	Human glomerular endothelial cells (GECs) + hiPSC-derived/immortalized podocytes	Demonstrated bidirectional cellular crosstalk and foot process formation; size-selective permeability (10/70 kDa dextrans).	Disease modeling (e.g., DN, FSGS, Alport syndrome); nanomedicine renal clearance assessment.	[232]
	Modeled immune-mediated glomerulonephritis on-chip using patient sera to induce barrier dysfunction.	Custom triple-chamber microfluidic chip; PET porous membrane (0.4 μm) separating cell layers.	Immortalized human podocytes (CIHP-1) + RFP-labeled human glomerular endothelial cells	Sera from IgA nephropathy and MN patients specifically increased albumin permeability.	Glomerulonephritis subtype modeling; autoimmune pathology; personalized disease modeling.	[233]
Tubule	Developed a 3D flow-directed proximal tubule MPS that maintained functionality for up to 28 days.	Single-channel Nortis MPS; collagen I matrix, type IV collagen coating.	Human primary PTECs	Expression of γ-glutamyl transferase (GGT), glucose reabsorption, ammonia genesis, vitamin D metabolism.	Drug clearance and nephrotoxicity prediction; long-term toxicity studies; physiological emulation.	[3]
	Investigated flow-induced regulation of drug transporter expression and kinetics in a proximal tubule chip.	Commercial microfluidic system (Elveflow) and chips (μ-Slide, Beonchip); thin-layer collagen I coating.	Immortalized human renal proximal tubule cells (RPTECs/TERT1s)	Flow upregulated transporter expression (OCT2, MATE1); enhanced polarization and metabolite transport.	Cationic drug transport studies; drug-drug interactions (DDI); local pharmacokinetics (PK)/PD assessment.	[234]
	Enhanced proximal tubule barrier performance by co-culturing hiPSC-derived organoid cells with immortalized cells.	PDMS-based dual-channel microfluidic chip with PET membrane.	hiPSC-derived renal organoid PTECs + Immortalized RPTECs/TERT1s	Co-culture showed higher transporter expression, enhanced glucose/albumin reabsorption, and P-gp efflux.	Personalized nephrotoxicity testing; advanced in vitro model development.	[110]
	3D-bioprinted convoluted proximal tubules with enhanced physiological functionality compared to 2D models.	3D bioprinting of sacrificial Pluronic F127 ink; encapsulated in gelatin-fibrin hydrogel.	Immortalized RPTECs/TERT1s cells	Formed 3D tubules with open lumen; enhanced microvilli density, polarization, and function.	Nephrotoxicity assessment (e.g., cyclosporine A); tubular disease mechanisms.	[157]
	Utilized a human tubule-on-chip to reveal polymyxin B-induced nephrotoxicity via cholesterol biosynthesis pathway activation.	Single-channel Nortis MPS; collagen I/IV coating.	Human primary RPTECs	PMB induced KIM-1 expression, specific miRNA profiles, and activated cholesterol biosynthetic pathways.	Antibiotic nephrotoxicity mechanism; safety assessment of novel antibiotics (NAB739/741).	[235]
	Constructed a dual-layer co-culture model in an injection-molded polycarbonate chip for nephrotoxicity assessment.	Injection-molded polycarbonate chip with insertible PET membrane culture inserts.	Human primary PTECs RPTECs + HUVECs	Validated dose-dependent toxicity of cisplatin and gentamicin; detected KIM-1/NGAL biomarkers.	Preclinical drug nephrotoxicity screening; animal model alternative.	[236]
	Enabled high-throughput screening of drug-transporter interactions in a 3D microfluidic tubule model.	Mimetas OrganoPlate platform (384-well format); collagen I gel channel.	Conditionally immortalized human proximal tubule cells (ciPTEC-OAT1)	Expressed OAT1, OCT2, P-gp, MRP2/4; validated P-gp substrate efflux function.	Early-stage drug transporter interaction screening; high-throughput toxicity assessment.	[237]
	Generated a 3D tubule model with enhanced drug uptake from sorted kidney organoid-derived cells.	Multi-chip device (MCD); sacrificial fishing line template to create parallel channels in ECM.	hiPSC-derived kidney organoid-sorted PTECs	Significant upregulation of OCT2/OAT1/OAT3; increased sensitivity to cisplatin and aristolochic acid toxicity.	Personalized drug safety and efficacy testing; disease modeling.	[52]
Vascularized	Demonstrated anastomosis between endogenous organoid microvasculature and engineered macrovessels for perfusion.	PDMS dual-channel chip; channels embedded in gelatin-fibrin matrix.	hESC-derived kidney organoids + HUVECs	Established lumen-to-lumen anastomosis; perfusion of fluorescent dextrans and red blood cells.	Vascularized tissue engineering; kidney development and regeneration.	[151]
	3D-bioprinted a vascularized proximal tubule model to study albumin uptake and glucose reabsorption.	3D bioprinting of sacrificial ink; co-culture in gelatin-fibrin hydrogel.	Immortalized human PTECs RPTECs/TERT1s + Human primary GMECs	Confirmed vectorial albumin transport from tubule to vasculature; glucose reabsorption function.	Renal physiological function; diabetic nephropathy modeling; SGLT2 inhibitor mechanisms.	[156]
	Fabricated complex, perfusable organ-specific microvasculature in collagen gel via multiphoton laser ablation.	Multiphoton laser ablation of collagen hydrogel; laser-guided angiogenesis.	HUVECs; Human coronary artery smooth muscle cells (hCASMCs)	Created alveolar, brain, and glomerulus-specific capillary networks; supported RBC perfusion.	High-fidelity organ-on-a-chip models; thrombosis, inflammation, tumor angiogenesis studies.	[165]
Multi-Organ	Constructed a liver-kidney chip to study indirect nephrotoxicity of drug metabolites (e.g., Aflatoxin B1).	Commercial COP dual-chamber interconnected chip (ChipShop); integrated bubble traps.	Human hepatoma cells (HepG2) + Human embryonic kidney cells (HEK293)	Hepatic metabolism of AFB1 to toxic epoxide metabolites caused downstream renal damage.	Drug metabolite toxicity assessment; liver-kidney inter-organ cross-talk.	[238]
	Developed a gut-kidney chip to study post-absorption nephrotoxicity and drug interactions.	PDMS bilayer chip; polycarbonate membrane separating gut and kidney compartments; Matrigel coating.	Human colon adenocarcinoma cells (Caco-2) + Rat primary glomerular endothelial cells	Validated Digoxin absorption and its interaction with Cholestyramine/Verapamil.	Integrated assessment of oral drug absorption and nephrotoxicity; drug-drug interactions.	[239]
Other	Developed a high-throughput chip array with integrated optical oxygen sensors for real-time metabolic monitoring.	COP chip array; integrated PyroScience oxygen sensor spots.	Human primary RPTECs	Real-time monitoring of oxygen consumption rate (OCR); detected drug-induced metabolic shifts (FCCP, antimycin A).	High-throughput drug metabolic toxicity screening; cellular metabolic function studies.	[107]
	Validated the feasibility of an implantable silicon nanopore membrane (SNM) bioreactor for renal cell therapy.	Silicon nanopore membrane (SNM) bioreactor; PEG surface modification for anti-thrombogenicity.	Human renal epithelial cells (HRECs)	Implanted in pigs for 7 days with >90 % cell viability and no hyperacute rejection.	Bioartificial kidney development; renal cell therapy; implantable medical devices.	[166]

Collectively, Accurate simulation and coupled control of the relevant physical, chemical and biological microenvironment represent the core technologies that distinguish renal barrier organoids from static models, enabling high-fidelity physiological/pathological reproduction. This not only brings cell behavior closer to the in vivo state but also provides unprecedented possibilities for dynamically studying the real-time response, damage mechanisms, and repair processes of barrier function under controllable stimulus conditions, such as drug exposure, hyperglycemia, hypoxia, and inflammatory factors. This capability forms the cornerstone for applications in precision drug evaluation and disease mechanism elucidation. However, achieving accurate and complex multi-parameter coupled regulation remains a significant technical challenge.

## 4 Evaluation metrics for renal barrier models: multidimensional validation of bionic functionality

The ultimate goal in constructing renal barrier OoC models is to accurately mimic their physiopathological functions. Therefore, establishing a systematic and multidimensional evaluation framework is paramount. This framework must rigorously verify structural biomimicry, passive barrier function integrity, active transport capabilities, and metabolic and secretory functions across multiple dimensions. A core advantage of OoC platforms is their inherent support for real-time, dynamic, and quantitative monitoring, thereby surpassing the limitations inherent to traditional static models and animal models.

### 4.1 Structural biomimicry and cellular state assessment

Validating the similarity of the OoC model to the in vivo renal barrier in terms of cellular morphology, tissue architecture, and expression of key molecules forms the essential foundation for functional reliability. This assessment primarily combines high-resolution imaging techniques with molecular biology methods, and OoC systems offer unique advantages in preserving and visualizing physiological cellular phenotypes through biomimetic microenvironment simulation.

#### 4.1.1 Cell morphology and ultrastructure

Immunofluorescence staining is employed to assess specific cellular features: podocyte foot processes are labeled with antibodies against Synaptopodin or Podocin [233], tight junctions are identified using markers like ZO-1 or Claudins [240], and tubular cell polarity is evaluated via staining for Na^+^/K^+^-ATPase (basolateral localization) or P-glycoprotein (P-gp, apical localization) [99,241,242]. Beyond conventional fluorescence microscopy, confocal laser scanning microscopy (CLSM) enables 3D reconstruction of cellular networks, revealing the spatial arrangement of podocyte foot processes or tubular microvilli and their interactions with neighboring cells [243,244]—an capability unavailable in 2D culture models. For ultrastructural analysis, transmission electron microscopy (TEM) is indispensable for visualizing subcellular details: it can resolve the 4–6 nm thick slit diaphragm between podocyte foot processes, the fenestrations (70–100 nm) in glomerular endothelial cells, and the trilaminar structure of the GBM [[245], [246], [247]]. Scanning electron microscopy (SEM) complements TEM by providing 3D surface topography, such as the density of tubular brush border microvilli or the integrity of endothelial fenestral networks [248].

The mechanistic basis underlying structural biomimicry in OoC models lies in the synergistic regulation of physical cues, biochemical signals, and cell-cell crosstalk. For example, physiological shear stress (10–20 dyn/cm^2^ for glomerular endothelium) induces cytoskeletal rearrangement via mechanosensitive ion channels (e.g., Piezo1), promoting the formation of endothelial fenestrations and podocyte foot processes [249,250]. ECM components like laminin and type IV collagen in the GBM mimicry layer bind to integrins on podocytes, activating focal adhesion kinase (FAK) signaling to stabilize foot process architecture [251]. In contrast, traditional 2D cultures lack these dynamic cues, leading to flattened podocytes with underdeveloped foot processes and disorganized tight junctions. Animal models, while recapitulating in vivo structures, suffer from species-specific differences (e.g., rodent podocytes have fewer foot process interdigitations than humans) and limited accessibility for real-time imaging of ultrastructural changes.

**Fig. 4:**
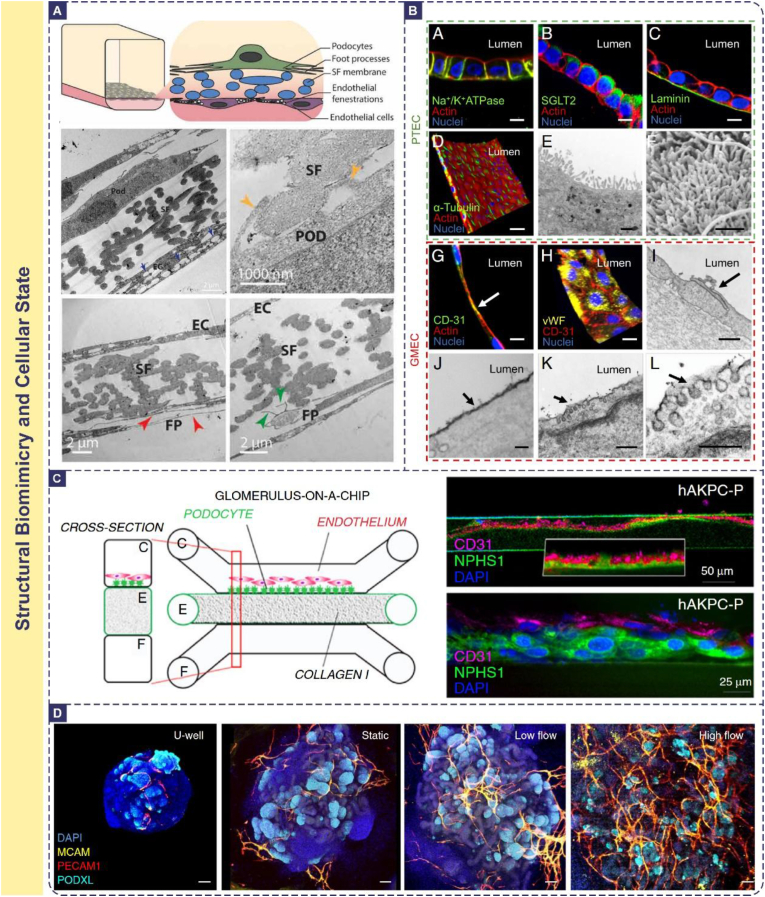
Evaluation and Validation of Renal Barrier Models. (A) High-resolution electron microscopy analysis of tissue-specific phenotypes in the engineered glomerulus biomimetic microfluidic device [98]. Red arrows indicate podocyte foot processes and formation of interdigitation-like organization, green arrows indicate formation of secondary and tertiary foot processes, and orange arrows indicate formation of short protrusions around the SF nanofibers. (B) PTECs and GMECs seeded in 3D VasPT tissues exhibit healthy and mature phenotypes. TEM and SEM micrographs showing densely packed PTEC microvilli that are ∼1.2 μm in height [156]. (C) The podocyte lines form a continuous layer, distinguishable from the human glomerular endothelial cells layer in Organoplate™ [16]. (D) Developing kidney organoids cultured in vitro under high fluid flow exhibit enhanced vascularization during nephrogenesis [186].

OoC platforms address these limitations by integrating biomimetic microenvironments with high-resolution imaging compatibility. For instance, Mou et al. directly observed the formation of complex secondary and tertiary foot processes and slit diaphragm-like structures in hiPSC-derived podocytes within the chip via TEM, confirming the model's capability to induce a highly mature phenotype with in vivo relevance(Fig. 4A) [98]. Homan et al. demonstrated that 3D bioprinted convoluted tubules, under physiological shear stress, exhibited a 3-fold increase in brush border microvilli density compared to static 2D models, as visualized by SEM [157]. These examples highlight the OoC's unique ability to preserve and visualize physiologically relevant ultrastructures by recapitulating the in vivo regulatory mechanisms of cell morphology.

#### 4.1.2 Expression and localization of key molecules

Quantitative techniques including quantitative real-time polymerase chain reaction (qPCR), Western blotting, and immunofluorescence are utilized to measure the expression levels of proteins crucial for barrier function, such as nephrin, podocin, VE-cadherin (endothelial junctions), aquaporin-2 (AQP2, water transport), and (SGLT2, nutrient transport). More critically, the spatial localization of these molecules—a determinant of barrier function—must be validated: for example, nephrin and podocin must colocalize at the podocyte cell membrane to form functional slit diaphragms [252], while Na^+^/K^+^-ATPase must be restricted to the basolateral membrane of tubular epithelial cells to establish the electrochemical gradient for active transport [253,254].

The mechanism linking molecular expression/localization to barrier function is rooted in signaling cascades modulated by the OoC microenvironment. For glomerular models, vascular endothelial growth factor (VEGF) secreted by podocytes binds to VEGFR2 on endothelial cells, upregulating the expression of endothelial nitric oxide synthase (eNOS) and promoting the formation of fenestrations [255]. In tubular models, shear stress activates the ERK1/2 pathway, enhancing the expression and apical localization of SGLT2, thereby boosting glucose reabsorption [256]. Key molecules often function in complexes: nephrin interacts with podocin to stabilize the slit diaphragm, and their mislocalization (e.g., cytoplasmic retention) in response to high glucose or toxins directly impairs filtration barrier integrity [252,257].

Compared to traditional models, OoCs offer distinct advantages in studying molecular dynamics. Static 2D cultures fail to induce the expression of mature barrier markers (e.g., nephrin expression is 2–3 fold lower than in vivo) [258,259], while animal models are impractical for real-time tracking of molecular localization changes [260]. OoCs enable dynamic monitoring of molecular expression and localization under controlled stimuli: for example, Zhang et al. used their glomerulus-proximal tubule chip to show that cisplatin exposure induces the cytoplasmic translocation of ZO-1 in tubular cells, coinciding with TEER reduction—an observation that directly links molecular mislocalization to barrier dysfunction [261]. Petrosyan et al.’s Glomerulus-On-A-Chip (GOAC) confirmed stable expression of podocyte markers (nephrin, WT1) for over 28 days, a feat unattainable in static cultures(Fig. 4C) [16].

#### 4.1.3 Degree of organization and vascularization

For models incorporating 3D structures or organoids, evaluating the degree of tissue organization, manifested by the formation of recognizable glomerular- or tubule-like structures, and the level of vascularization, indicated by vascular network density, branching complexity, and perfusability, is essential [151,262]. The mechanism driving tissue organization in OoCs is the recapitulation of in vivo cell-cell and cell-ECM crosstalk: renal organoids in OoCs self-organize into glomerular-like structures with podocytes, endothelial cells, and mesangial cells due to paracrine signaling (e.g., Ang-1/Tie2) and ECM-guided cell migration [263,264]. Vascularization is promoted by fluid shear stress, which upregulates VEGF and angiopoietin expression, facilitating the anastomosis of organoid-derived microvessels with chip-based macrochannels [265].

OoCs outperform traditional 3D cultures in vascularization: Homan et al. achieved a 5-fold increase in PECAM1-positive vascular area and a 10-fold increase in connection density in perfused kidney organoids compared to static organoid cultures(Fig. 4D) [186]. Kroll et al.’s vascularized organoid chip demonstrated functional perfusion of red blood cells through the organoid microvessels, a critical milestone for simulating in vivo nutrient and waste exchange [151]. Animal models, while vascularized, cannot be dissected to study the molecular mechanisms of vascular-tubular crosstalk without disrupting tissue integrity, whereas OoCs allow for real-time imaging of vascular-endothelial-tubular interactions.

To systematically evaluate the specific molecular expression of key cell types and structures, Table 4 summarizes the critical markers commonly used for validating the biomimicry of renal barrier chip models and their functional significance.

**Table 4:** Key renal barrier markers.

	Marker	Target of Marker	Localization/Expressing Cells	Staining Method	Functional Significance	Ref
Glomerular	Nephrin	Slit diaphragm protein	Podocytes	Immunofluorescence	Core structure of the filtration barrier; deficiency causes proteinuria	[102]
	Collagen IV (Col IV)	GBM	GBM/mesangial matrix	Immunofluorescence	Main component of GBM; thickening indicates diabetic nephropathy	[186]
	CD31/PECAM1	Endothelial cell junction	Glomerular endothelial cells	Immunofluorescence	Evaluation of vascular integrity; high expression in chips indicates maturity	[16,186]
	WT1	Podocyte nucleus	Podocytes	Immunohistochemistry	Marker of podocyte differentiation; deficiency indicates detachment	[102]
Renal Tubular	LTL/FITC	Brush border polysaccharide	Proximal tubular epithelium	Chemical fluorescence staining	Marks proximal tubule segments; enhanced expression in flow culture	[186,293]
	Megalin	Apical membrane endocytic receptor	Proximal tubular epithelium	Immunofluorescence	Mediates protein reabsorption; detached upon injury	[294]
	AQP1	Aquaporin	Proximal tubule/descending limb of Henle's loop	Immunofluorescence	Key to water reabsorption; deficiency causes polyuria	(Fig. 5C) [295]
	ZO-1	Tight junction	Apical side of tubular epithelium	Immunofluorescence	Core of barrier integrity; high expression is positively correlated with TEER	[3,296]
	KIM-1	Transmembrane glycoprotein	Injured proximal tubules	Immunofluorescence	Early marker of acute injury; evaluation of drug toxicity	[295]
	E-cadherin	Intercellular junction	Distal tubular epithelium	Immunofluorescence	Maintains polarity; deficiency promotes fibrosis	[3,195]
	Calbindin	Calcium-binding protein	Distal tubules	Immunofluorescence	Marker of calcium reabsorption function	[297]
Collecting Duct	AQP2	Aquaporin	Principal cells of collecting duct	Immunofluorescence	Regulates urine concentration via ADH; mutation causes diabetes insipidus	[15]
	Na^+^/K^+^-ATPase	Basolateral membrane	Principal cells of collecting duct	Immunofluorescence	Maintains sodium-potassium gradient	[234]
Kidney-Wide Applicable	DAPI	Nuclear DNA	Cell nucleus	Chemical fluorescence staining	Reference for nuclear localization; assists in cell counting/morphological observation	[186,293]
	PAS Staining	Basement membrane/glycogen	GBM/tubular basement membrane	Histochemical staining	Evaluates basement membrane thickening or tubular atrophy	[296]
	α-SMA	Myofibroblasts	Fibrotic areas	Immunohistochemistry	Marker of epithelial-mesenchymal transition (EMT); predicts fibrosis	[296]
	Phalloidin	F-actin	Cytoskeleton	Chemical fluorescence staining	Observes cell morphology and polarity	[16,295]

### 4.2 Passive barrier functional integrity assessment

#### 4.2.1 Trans-epithelial/endothelial electrical resistance (TEER)

The core of barrier functional integrity assessment involves quantifying the selective permeability of the barrier to substances, reflecting its physical sieving and charge barrier functions. The OoC platform uniquely supports real-time monitoring within a dynamic fluidic environment, enabling the study of barrier function under physiological conditions.

TEER serves as the gold standard metric for assessing the tightness of epithelial or endothelial barriers, with values directly correlated to the integrity of tight junctions and cell monolayer confluency [207,266]. It is dynamically measured in real-time using integrated microelectrodes in OoCs, providing continuous data on barrier stability over days or weeks [267,268]. The mechanistic basis of TEER is the resistance to ion flow across the cell monolayer: tight junction proteins (ZO-1, occludin, claudins) form a seal between cells, and their expression level and phosphorylation state directly modulate ion permeability [269]. For example, claudin-16 and claudin-19 form cation-selective pores in the thick ascending limb, contributing to TEER values specific to that nephron segment [270].

**Fig. 5:**
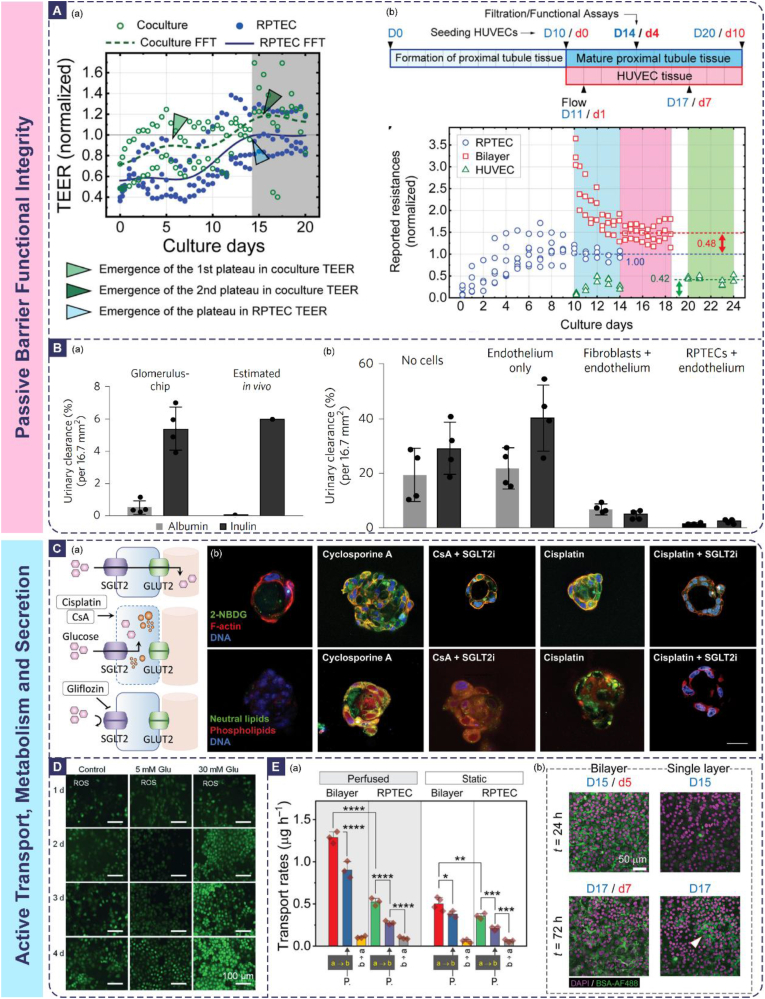
(A) Co-culturing hiPSC-derived tubular cells with immortalized cells in a flow-based OoC resulted in a 2-fold higher TEER compared to static co-cultures(a) Evaluation by the TEER evolution with culture time for the RPTEC-only and coculture tissue layers. (b) Time course of reported resistances of the RPTEC-only (blue circles), bilayer (red squares), and HUVEC-only (green triangles) tissue layers [110].(B) Microfluidic organ-on-a-chip device reconstitutes kidney glomerular capillary function in vitro. (a) Quantification of the glomerular filtration (urinary clearance) of albumin and inulin molecules that were continuously infused over 6 h into the capillary channel of the glomerulus chip that was lined by hiPS-cell-derived podocytes and human glomerular endothelial cells. (b) Filtration of albumin and inulin in control microfluidic chips without human kidney podocytes quantified over 6 h of continuous infusion using the methods described above. RPTECs, renal proximal tubular epithelial cells [102]. (C) Cyclosporine and cisplatin toxicity are reversed by SGLT2 inhibition. (a) Schematic of glucose transport in proximal tubule cells and mechanism of nephroprotective effect of empagliflozin (gliflozin). (b) Fluorescent glucose analog and lipid accumulation in 3D cysts exposed to cyclosporine or cisplatin in the presence or absence of empagliflozin [295]. (D) Reactive oxygen species production of glomerular cells after exposure to high glucose conditions at different concentrations [100]. (E) Assessment of the filtration capacity of RPTEC tissue showing the effects of HUVECs and flow induced shear stress. (a) Transfer rates of the glucose probe, 2NBDG measured in static and perfused culture conditions. Both reabsorption (a → b) and reverse transfer rates (b → a) were quantified. (b) Fluorescent confocal z-stacked images of the RPTEC tissue layer in bilayer and single layer configurations. A considerably higher amount of BSA was precipitated in the basolateral milieu of RPTECs in the bilayer system indicating a higher intake of the substrate [110].

OoCs offer significant advantages over traditional models in TEER measurement: static 2D cultures provide only endpoint TEER values and fail to account for dynamic changes induced by shear stress or biochemical gradients. Animal models cannot measure TEER in situ without invasive procedures that disrupt tissue function. OoCs enable real-time TEER monitoring under physiological stimuli: Sadeghian et al. showed that co-culturing hiPSC-derived tubular cells with immortalized cells in a flow-based OoC resulted in a 2-fold higher TEER compared to static co-cultures, attributed to shear stress-induced upregulation of tight junction proteins(Fig. 5A) [110]. This dynamic measurement capability allows for the study of barrier disruption and recovery (e.g., after drug-induced injury), a critical feature for toxicity testing.

#### 4.2.2 Molecular selective permeability

This metric evaluates the barrier's ability to discriminate between molecules based on size and charge, a core function of both the GFB and tubular tight junctions. Under simulated physiological flow fields and pressures, fluorescently or radiolabeled tracer molecules of varying sizes (e.g., inulin, 5.5 kDa; albumin, 66 kDa; IgG, 150 kDa) and charges (e.g., neutral dextran versus negatively charged albumin) are perfused into the "blood"/"lumen" side, and their translocation to the opposing side is quantified [100,199].

For successful glomerular models, high retention of large proteins is essential, exemplified by albumin retention exceeding 99 %, while free filtration of small molecules should occur, with inulin filtration rates typically around 5 % or 7 %(Fig. 5B) [16,98,102]. The spherical hollow-fiber chip of Dai et al. successfully validated comparable selective filtration properties [108]. For renal tubule models, their ability to block passive diffusion of small molecular solutes was assessed.

The mechanistic basis of selective permeability lies in the dual barrier properties of the renal barrier: size selectivity is determined by the GBM's pore size (<4 nm) and slit diaphragm width (30–50 nm) [271,272], while charge selectivity is mediated by negatively charged molecules (e.g., heparan sulfate proteoglycans in the GBM and podocyte glycocalyx) that repel anionic proteins like albumin [273,274]. In tubular barriers, tight junctions restrict the paracellular diffusion of small solutes, with permeability regulated by claudin isoforms (e.g., claudin-2 forms water-permeable pores in the proximal tubule) [275,276].

OoCs surpass traditional models in recapitulating selective permeability: static 2D cultures lack the 3D structure and dynamic cues required for proper barrier formation, leading to overestimated permeability to macromolecules. Animal models exhibit species differences in barrier properties (e.g., rodent GBM has a higher pore density than human GBM), limiting translational relevance [187,277]. OoCs achieve physiological levels of selective permeability: Lin et al. confirmed vectorial albumin transport from the tubular lumen to the adjacent vasculature, directly demonstrating the barrier's capacity for selective protein handling and reabsorption [156]. Kim et al. developed a tubule-on-a-chip model which exhibited significantly upregulated expression of organic anion and cation transporters (OAT1/3, OCT2), leading to improved, physiologically relevant drug uptake and enhanced sensitivity to nephrotoxins [236]. These results demonstrate the OoC's ability to recapitulate the molecular mechanisms of selective permeability, making it a reliable tool for studying barrier dysfunction in diseases like diabetic nephropathy (where GBM thickening reduces size selectivity) or Alport syndrome (where GBM charge barrier is impaired).

### 4.3 Active transport, metabolism and secretion assessment

Beyond static barrier assessment, the focus of functional validation lies on the active cellular reabsorption and secretion of solutes in a dynamic microenvironment, as well as basal energy metabolism and renal-specific synthesis or clearance functions. This focused functional assessment represents a critical validation that distinguishes OoC models from passive barrier assessments. OoCs enable the study of these processes under dynamic, physiologically relevant conditions, providing insights into their regulatory mechanisms.

#### 4.3.1 Active transport function

Monitoring the active transport function of the barrier encompasses several perspectives. Ion transport is assessed using microelectrodes or fluorescent probes to monitor trans-epithelial gradients of Na^+^, K^+^, or H^+^, reflecting the activity of ion channels (e.g., ENaC) and pumps (e.g., Na^+^/K^+^-ATPase, H^+^-ATPase) [278,279]. Wang et al. model investigating viral infection examined associated sodium transport disorders [109]. Nutrient reabsorption (e.g., glucose, amino acids) is quantified by measuring the uptake of radiolabeled or fluorescently labeled substrates from the tubular lumen [261]. Drug/solute secretion assays measure the efficiency of active transport for specific drugs, including cisplatin, cimetidine, and methotrexate, or endogenous substances like creatinine, the organic anion para-aminohippurate (PAH), and the organic cation tetraethylammonium (TEA), from the basolateral/interstitial side to the luminal side. Quantification is achieved by comparing concentration changes across the barrier or detecting accumulation in the luminal effluent [261,280].

OoCs offer unique advantages over traditional models for studying active transport: static 2D cultures lose cell polarity and transporter expression over time, leading to reduced transport activity. Animal models have species-specific transporter expression profiles (e.g., rodent OAT1 has different substrate affinity than human OAT1) and are difficult to use for high-throughput studies [281]. OoCs maintain cell polarity and transporter function for weeks: Weber et al.’s proximal tubule chip retained glucose reabsorption and ammonia genesis for 28 days, with SGLT2 expression levels comparable to human kidney tissue [3]. Petit et al.’s OoC showed that flow-induced shear stress upregulated OCT2 and MATE1 expression, enhancing cation transport—an effect not observed in static cultures [234]. These capabilities make OoCs ideal for studying transporter-mediated drug-drug interactions and nephrotoxicity.

#### 4.3.2 Energy metabolism activity

Energy metabolism is a critical indicator of cellular functional status, as renal barrier cells (e.g., proximal tubule epithelial cells) have high energy demands for active transport. Glycolytic activity is assessed by measuring glucose consumption and lactate production in the culture medium, while mitochondrial respiration is evaluated by monitoring OCR using integrated optical sensors [282,283]. The mechanism linking metabolism to barrier function is that active transport (e.g., Na^+^/K^+^-ATPase) consumes ∼70 % of the kidney's ATP, and metabolic dysfunction directly impairs barrier function [284,285]. For example, cisplatin-induced mitochondrial damage reduces ATP production, leading to Na^+^/K^+^-ATPase inactivation and tubular barrier dysfunction [286,287].

OoCs outperform traditional models in metabolic monitoring: static 2D cultures have altered metabolic profiles due to nutrient gradients and lack of shear stress. Animal models cannot measure real-time metabolic changes in specific nephron segments. OoCs enable real-time, segment-specific metabolic monitoring: Kann et al.’s high-throughput OoC platform integrated oxygen sensors to detect drug-induced changes in OCR, identifying metabolic shifts induced by FCCP and antimycin A [107]. Dai et al. observed that flow-cultured glomerular cells had higher glucose consumption than static cultures, reflecting increased energy demand for maintaining barrier function [108].

#### 4.3.3 Synthesis and clearance functions

These functions indicate the functional maturity of renal barrier cells, including the metabolism of glutamine to ammonia (proximal tubule), activation of vitamin D (proximal tubule), urea synthesis, and secretion of erythropoietin (peritubular interstitial cells) and renin (juxtaglomerular cells) [3,108]. The mechanism of these processes is mediated by cell-specific enzymes and signaling pathways: for example, 1α-hydroxylase in proximal tubule cells converts 25-hydroxyvitamin D to the active form (1,25-dihydroxyvitamin D), regulated by parathyroid hormone (PTH) [288].

Assessment of barrier integrity can also be informed by detecting cellular damage markers. These include apoptosis/necrosis markers such as lactate dehydrogenase (LDH) release, elevated levels of ROS, and activation of caspase-3, as well as the expression of specific injury molecules like Kim-1 [100]. Assessment of barrier integrity is complemented by detecting cellular damage markers, including apoptosis/necrosis (LDH release, caspase-3 activation, ROS production) and specific injury molecules (e.g., KIM-1, NGAL) [289]. The mechanism of damage marker release is that cellular stress (e.g., drug toxicity, hypoxia) disrupts membrane integrity (leading to LDH release) or activates signaling pathways (e.g., NF-κB-mediated KIM-1 expression) [[290], [291], [292]]. OoCs enable real-time monitoring of these markers under controlled stimuli: Wang et al.’s diabetic nephropathy model showed increased ROS production and KIM-1 expression in response to high glucose, correlating with barrier permeability(Fig. 5D) [100]. This real-time correlation between damage markers and barrier function is unavailable in traditional models.

Integration of the aforementioned multidimensional metrics underscores a concerted move towards a standardized evaluation framework for renal OoCs. A consensus is emerging on essential quantitative benchmarks, such as physiological albumin retention and functional transporter activity, which are increasingly reported across studies. However, significant barriers to full standardization persist, primarily stemming from the inherent variability in platform materials, cell sourcing, and operational parameters. This lack of uniformity complicates direct inter-laboratory comparisons and remains a pivotal challenge for the quantitative validation and broader adoption of these models in translational pipelines.

A well-established renal barrier OoC evaluation system commences with the validation of static structural biomimicry. This evaluation system centers on the quantitative characterization of the barrier's key physiological functions under dynamic conditions. The pivotal advantage of OoC technology lies in its capacity to seamlessly integrate multiple sensing and imaging modalities, such as integrated TEER electrodes, optical oxygen/ion/pH sensors, and fluorescence microscopy. This integration enables real-time, dynamic, and potentially high-throughput monitoring of the aforementioned metrics within simulated physiological or pathological microenvironments. Consequently, OoC platforms provide an unprecedented high-fidelity toolset for the in-depth study of renal barrier physiology, precise elucidation of disease mechanisms, and accurate assessment of drug effects.

## 5 Application scenarios: core value that breaks through traditional limitations

The core value of renal barrier OoC technology lies in its successful circumvention of key bottlenecks inherent to traditional research models, such as animal experiments and static cell cultures. It provides a novel paradigm for tackling persistent challenges in kidney research, drug development, and precision medicine. It leverages advantages in biomimetic structure, dynamic microenvironment simulation, and real-time monitoring. Its transformative application value manifests in the following interconnected and groundbreaking directions, with irreplaceable potential particularly in personalized medicine and high-throughput drug research and development.

### 5.1 Dynamic simulation and personalized modeling of complex kidney disease mechanisms

Animal models often fail to accurately reproduce human disease-specific pathologies, such as the unique proteinuria features observed in human DKD. Static models cannot simulate dynamic microenvironmental changes occurring during disease progression, including sustained hyperglycemia, hypoxic gradients, or fluctuations in inflammatory factors. Furthermore, obtaining patient-specific models to study genetic disorders or individual variations remains difficult.

**Fig. 6:**
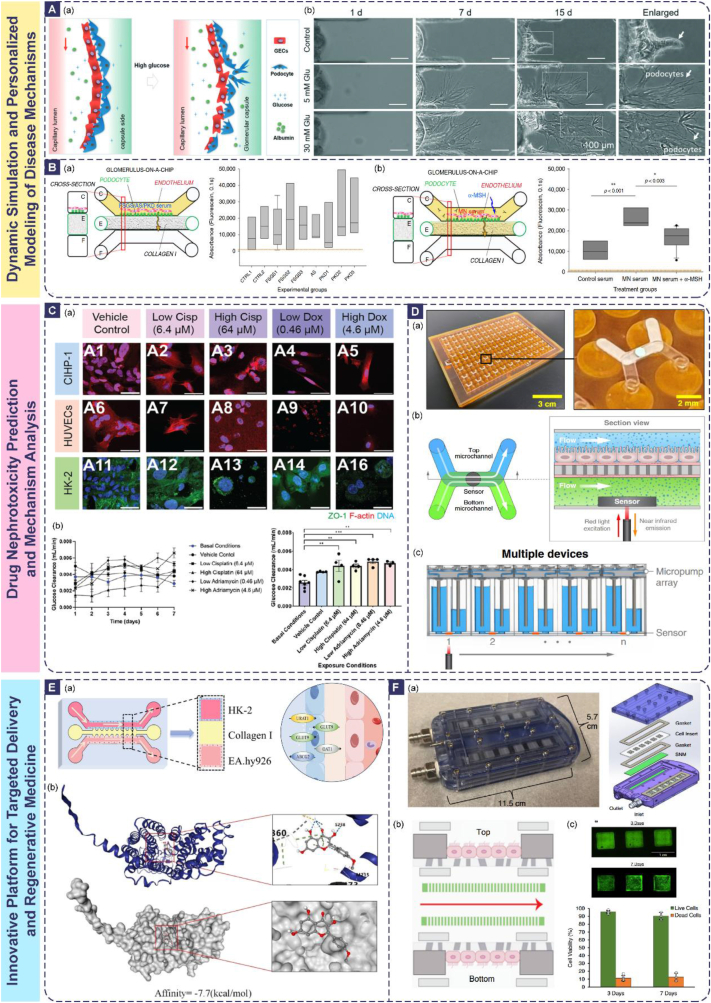
Representative Renal Barrier Applications. (A)A disease model of diabetic nephropathy in a glomerulus-on-a-chip microdevice. (a) GFB filtration dysfunction under high glucose conditions (b) Visualized images of migrated glomerular cells along with the GFB on 3D basement membrane in dynamic culture under high glucose conditions. The podocyte processes were observed to protrude into the 3D Matrigel over time. The white arrows represent the podocyte processes [100]. (B) Validation of the hAKPC-P GOAC system as a diagnostic and drug screening platform. (a) The GOAC was exposed to serum from patients with other kidney diseases (FSGS, AS, PKD) to assess albumin permeability. (b) The GOAC was treated with MN serum in the presence or absence of the therapeutic drug α-MSH, and its effect on albumin leakage was evaluated [16]. (C) Drug-induced nephrotoxicity tests of glomerulus and proximal tubule MPS. (a) Dynamic cellular images at 7 days after exposure to nephrotoxins in Drug-induced nephrotoxicity tests. (b) Glucose clearance (mL min−1) across all drug conditions [261]. (D) Measurement of oxygen consumption rates of human renal proximal tubule cells in an array of organ-on-chip devices to monitor drug-induced metabolic shifts. (a) Bottom view of the O-MCP with 96 devices and a zoomed-in image of a single device. (b) Cross-section side view of an O-MCP device. (c) The O-MCP and corresponding measurement technique allowed single devices to be measured repeatedly or multiple devices to be measured sequentially [107]. (E)Validation of targeted inhibition of URAT1 transporter by kaempferide based on a kidneyon-a-chip. a) Design and structure of the microfluidic chip.; b) 3D structure drawing and binding site prediction of the docking model of kaemperide and URAT1 [315]. (F)An implantable bioreactor for renal cell therapy. (a) Bioreactor design and constituent components. (b) Relative positional relationship between the blood flow pathway and the cell culture region. c) High cell viability on cell inserts after 3- and 7- day implants [166].

By accurately introducing and dynamically modulating pathological stimuli—such as sustained hyperglycemia or inflammatory factors—on a foundation of biomimetic structures, microfluidic chip technology enables the modeling of diverse renal pathological states [298]. For example, Wanget al. and Garreta et al. applied a high-glucose environment within glomerular biochips, successfully inducing key DKD phenotypes, including basement membrane thickening, increased podocyte detachment, elevated ROS levels, and enhanced albumin permeability(Fig. 6A) [100,293]. Crucially, OoC platforms allow for the dynamic observation and quantitative analysis of barrier dysfunction, metabolic abnormalities, gene expression alterations, and morphological pathologies triggered by these stimuli. Kroll et al. developed an immune-infiltrated organoid biochip that dynamically replicates T-cell-mediated renal injury in a dose-dependent manner upon sequential addition of immune cells and antibodies [99].

In the field of personalized medicine, combining iPSC with gene editing technologies has opened new avenues for precise modeling of hereditary kidney diseases. Musah et al. constructed glomerular chips using patient-derived or CRISPR-edited iPSC-differentiated podocytes. These platforms provide a humanized system for investigating molecular sieve dysfunction in hereditary disorders like those caused by NPHS mutations, which can directly reflect patient-specific pathological characteristics. [102,103]. Li et al. employed CRISPR to generate polycystic kidney disease (PKD) mutant organoid biochips, innovatively revealing a novel mechanism where cyst expansion is primarily driven by glucose uptake, thereby providing a target validation platform for tailoring personalized treatment plans for patients [296]. For complex diseases such as diabetic nephropathy, researchers can collect peripheral blood or urine cells from different patients to induce iPSCs, which are then differentiated into glomerular endothelial cells, podocytes, and renal tubular epithelial cells to construct patient-specific chip models [299]. These models can simulate individual differences in tolerance to risk factors such as hyperglycemia and hypertension, predict disease progression rate, and evaluate responses to hypoglycemic and hypotensive drugs [300]. The GOAC system reported by Petrosyan et al. can recapitulate patient-specific proteinuria when perfused with patient serum, and the degree of albumin leakage is positively correlated with the severity of individual disease—providing a quantitative tool for dynamically assessing disease activity and personalized treatment efficacy(Fig. 6B) [16].

Simultaneously, microfluidic chips support long-term culture and real-time monitoring, facilitating the tracking of dynamic pathological processes. For instance, in hereditary nephritis models, long-term observation of changes in the expression of podocyte slit diaphragm proteins (e.g., nephrin, podocin) and progressive increases in basement membrane thickness via chips provides a research tool that traditional models cannot achieve for deciphering the time-dependent mechanisms of disease progression [301].

### 5.2 Drug nephrotoxicity prediction and mechanism analysis

Species differences in animal models frequently lead to inaccurate toxicity predictions; for instance, cisplatin exhibits greater tolerance in mice than in humans [302,303]. Static cellular models not only fail to simulate the dynamic processes of drug exposure in vivo—such as concentration gradients and clearance rates—but also struggle to capture the influence of hemodynamics on cellular responses, complicating the assessment of secondary toxicity from metabolites. OoC technology has achieved breakthrough improvements in the accuracy, sensitivity, and mechanistic analysis depth of drug nephrotoxicity assessment by dynamically simulating the physiological microenvironment.

**Dynamic Exposure and Physiological Clearance Simulation:** Microfluidic systems enable precise control over drug concentration, exposure duration, and clearance rate, thereby simulating human PK. Kim et al. successfully reproduced the distinct pharmacokinetic profiles of single bolus versus continuous infusion regimens of gentamicin on a renal tubule biochip. The study found the single bolus regimen induced lower nephrotoxicity, providing direct evidence to support optimizing clinical dosing schedules [101]. For drugs requiring hepatic metabolic activation (e.g., certain chemotherapeutic agents), liver-kidney coupled chip platforms can be constructed to simulate the in vivo process of "metabolism-dependent nephrotoxicity" through toxic intermediate products generated by hepatocyte metabolism acting on renal cells, avoiding underestimation of toxicity in traditional single renal cell models due to the lack of metabolic links [304,305].

**High-Throughput Screening and Automated Analysis:** Traditional renal toxicity screening relies on 2D cell models, which have low throughput and poor predictability. However, OoC technology enables high-throughput, multi-parameter toxicity assessment through miniaturization, array design, and integration with automated systems. For instance, the renal chip array based on the Mimetas OrganoPlate® platform (with expandable 384-well throughput) has been utilized for nephrotoxicity screening of dozens of drugs [306,307]: fluid shear stress and renal tubular cell polarity are maintained via an automated perfusion system, while high-content imaging is integrated to quantitatively analyze cell viability, ZO-1-mediated tight junction integrity, and the release of toxicity biomarkers such as KIM-1 and NGAL [308,309]. Automated renal chip systems integrated with artificial intelligence (AI) have become a key development direction in the industry: the integrated platform developed by Emulate Inc. incorporates real-time TEER monitoring, ROS/apoptosis fluorescent probe detection, and a machine learning-based data analysis module, enabling rapid nephrotoxicity classification of compounds [310]. Its accuracy in predicting safe doses in humans is 35 %–45 % higher than that of traditional 2D models, and it has been adopted by pharmaceutical companies for optimizing preclinical screening processes [200,311].

In terms of mechanism analysis, high-throughput chip platforms can combine multi-omics technologies (transcriptomics, proteomics) to reveal the molecular mechanisms of drug toxicity. For example, Weber et al. studied the nephrotoxicity of polymyxin B using a proximal tubular chip and found that the drug induces tubular injury by activating the cholesterol biosynthesis pathway, rather than direct cell necrosis as traditionally thought—providing a new target for developing toxicity inhibitors [235]. Kann et al. developed a high-throughput organ-on-chip platform that enables label-free detection of metabolic shifts by exposing cells to drugs such as FCCP (which increases oxygen consumption), as well as antimycin A and oligomycin (which decrease oxygen consumption)(Fig. 6D) [107]. The integration of AI technology further improves screening efficiency: by training machine learning models based on chip data, the nephrotoxicity risk of compounds and potential targets can be quickly predicted [312,313]. For instance, by analyzing the similarity between drug-induced TEER changes, metabolomic characteristics, and known nephrotoxic drugs, toxicity grading and preliminary mechanism judgment of unknown compounds can be realized.

**Real-Time Multi-Barrier Function Monitoring:** Under simulated physiological flow conditions, OoC platforms enable real-time monitoring of drug effects on barrier integrity (e.g., TEER decrease), selective filtration (e.g., increased albumin leakage), transport function (e.g., inhibition of glucose reabsorption), and cellular viability (e.g., LDH release, elevated ROS levels, and caspase-3 activation). This capability allows the capture of early and sensitive toxicity signals [100]. Cohen et al. identified that cyclosporine can disrupt cellular polarity and induce lipotoxicity at very low concentrations on proximal tubule chips—a phenomenon not observed in static cultures due to the lack of shear stress stimulation [295]. The integrated glomerulus-proximal tubule chip of Zhang et al. successfully recapitulated the specific impairments of cisplatin and doxorubicin on filtration, reabsorption, and secretion functions, providing a tool for evaluating the organ region-specific toxicity of drugs(Fig. 6C) [261].

**Metabolite Toxicity Assessment:** Liver-kidney coupled OoC platforms address a critical gap in toxicity testing. The liver-kidney biochip study of Theobald et al. demonstrated that hepatocytes metabolize aflatoxin B_1_ (AFB_1_) and Benzo(a)pyrene (BαP) into toxic epoxide metabolites, causing damage to downstream renal cells. This directly elucidated the mechanism of metabolism-mediated nephrotoxicity, which cannot be detected by traditional single renal cell models [238].

### 5.3 Innovative platform for targeted delivery and regenerative medicine

Conventional models struggle to effectively evaluate the efficiency and targeting of nanocarriers traversing complex biological barriers. In vitro expansion of functional renal cells is challenging, and validating their therapeutic potential within a simulated physiological environment remains difficult. Renal barrier-on-a-chip technology provides a high-fidelity evaluation platform for the optimization of targeted delivery systems and regenerative medicine research.

**Renal Barrier Penetration and Targeting Assessment of Nanomedicines:** OoC platforms allow this assessment under simulated physiological blood flow and barrier structures. By quantitatively analyzing factors like nanoparticle size, charge, and surface modification, researchers can uncover the principles governing their efficiency in penetrating the GFBor targeting tubular/podocyte cells [233,314]. Song et al. using a dual-channel renal biochip integrating human renal tubular epithelial cells (HK-2) and vascular endothelial cells (EA.hy926), demonstrated that kaempferol (kaemperide), by targeting and inhibiting the URAT1 transporter, significantly reduced vascular channel uric acid concentrations and mitigated kidney injury—providing direct evidence for the screening and mechanism verification of targeted drugs for hyperuricemia(Fig. 6E) [315]. For nanodrugs used in kidney disease treatment (e.g., liposomes loaded with hormones or immunosuppressants), chips can be used to evaluate their targeted enrichment efficiency under pathological conditions (e.g., increased barrier permeability mediated by inflammation), optimizing carrier design to improve drug concentration at lesion sites and reduce systemic side effects [316,317].

**Validation for Cell Therapy and Bioartificial Organs:** This technology can provide a dynamic microenvironment closely resembling the in vivo setting for functional renal cells (whether iPSC-differentiated or primary), enabling the validation of their therapeutic potential. For instance, The implantable silicon nanopore membrane bioreactor of Kim et al. successfully encapsulated and maintained the viability and function of human renal epithelial cells (survival rate >90 %, no rejection) for 7 days in a porcine model, demonstrating the feasibility of bioartificial kidneys(Fig. 6F) [166]. Furthermore, the vascularized organoid biochips developed by Homan et al. and Kroll et al. established the foundation for constructing engineered renal tissues with perfusion capability, which is expected to solve the vascularization problem of tissue-engineered kidneys [151,186]. In cell therapy evaluation, iPSC-derived renal progenitor cells can be seeded into the renal interstitium-mimicking region of the chip to monitor their migration, differentiation, and repair effects towards damaged renal tubules in a dynamic microenvironment—providing safety and efficacy data for the clinical translation of cell therapy [245,318].

The application scope of renal barrier OoCs has expanded significantly from single toxicity tests to becoming a powerful engine driving precision drug safety evaluation, deep mechanistic analysis of human diseases, and the development of innovative therapies, including targeted delivery and cell/tissue-based treatments. Their core value resides in building a high-fidelity bridge between basic research and clinical translation through biomimetic structures, dynamic microenvironment simulation, real-time functional monitoring, and patient-specific cell integration. The successful cases highlighted in the literature include predicting the optimal nephrotoxicity-sparing dosing regimen for gentamicin, revealing the novel glucose uptake-driven mechanism in PKD cyst expansion, and validating the feasibility of implantable bioreactors. These cases compellingly demonstrate the transformative potential of this technology in solving intractable problems inherent to traditional research paradigms. As the technology matures further, particularly through multi-organ integration, enhanced long-term stability, and standardization, its value in advancing renal precision medicine and efficient drug discovery is poised to become even more prominent.

## 6 Challenges and future directions

The development of renal barrier OoC technology has reached a pivotal stage, demonstrating immense potential while concurrently exposing critical bottlenecks that hinder its full maturation and translational application. A thorough and critical analysis of these challenges is essential to guide future research. This section expands upon the current discourse to address the multifaceted limitations as highlighted by the research community, outlining a structured pathway for innovation.

**Material Challenges: Beyond Biocompatibility to Functional Biomimicry.** The reliance on polymers like PDMS, while facilitating rapid prototyping, introduces significant drawbacks that compromise model fidelity. Key issues include: (1) Poor intrinsic cell adhesion, necessitating extensive surface modifications (e.g., oxygen plasma treatment, protein coating) which can be unstable over time; (2) Leaching of uncured oligomers, which can exert unforeseen cytotoxic effects and interfere with drug response assays; (3) High absorption of small hydrophobic molecules and hormones, leading to inaccurate pharmacokinetic and pharmacodynamic data; and (4) Inherent instability under long-term perfusion, including potential swelling, deformation, or delamination of bonded layers. Furthermore, materials like PMMA and PC, while optically superior and less absorptive, are often rigid and non-degradable, failing to replicate the dynamic, remodelable nature of the native extracellular matrix. The future lies in developing next-generation biomaterials. This includes the synthesis of advanced, non-absorptive elastomers (e.g., perfluoropolymers, acrylic-based resins), the refinement of naturally derived hydrogels (e.g., silk fibroin, chitosan, hyaluronic acid) with tunable mechanical and degradative properties, and the creation of bio-inks for 3D printing that incorporate bioactive motifs (e.g., RGD, IKVAV) to enhance cell adhesion and function without the need for post-processing.

**Cell Source Limitations: The Quest for Physiological Relevance and Personalization.** A persistent bottleneck is the scarcity of reliable, functionally mature, and phenotypically stable human renal cells. Primary human renal cells are difficult to procure, exhibit limited expandability, and rapidly dedifferentiate in vitro. While immortalized cell lines offer scalability, they often harbor aberrant genetic and functional characteristics. iPSC-derived renal cells represent a revolutionary pathway, enabling patient-specific disease modeling. However, significant challenges remain: (1) Incomplete maturation, where iPSC-derived podocytes frequently fail to form intricate, stable foot processes and slit diaphragms, and tubular cells may not achieve full polarization and transporter expression profiles; (2) Batch-to-batch variability in differentiation efficiency; and (3) Lack of key stromal and immune components (e.g., mesangial cells, resident macrophages) that are crucial for creating a holistic tissue microenvironment. Future strategies must focus on optimizing differentiation protocols via combinatorial growth factor signaling and small molecule induction, employing CRISPR/Cas9 for precise genetic engineering of disease-associated variants, and developing robust protocols for the co-differentiation and co-culture of multiple renal cell types within a single system to recapitulate the cellular complexity of the nephron.

**Fabrication and Resolution Constraints: Pushing the Boundaries of Microfabrication.** While soft lithography and extrusion-based 3D bioprinting have been foundational, they face inherent resolution limits that are inadequate for replicating critical submicron features of the renal barrier [250], such as the slit diaphragm or the dense, uniform pore structure of the GBM. Furthermore, challenges in feature fidelity, structural stability of soft hydrogels under flow, and biocompatibility of fabrication processes (e.g., photoinitiator toxicity in vat polymerization) remain. Emerging technologies are poised to address these gaps. Multiphoton laser ablation and polymerization allow for the direct, maskless writing of complex 3D microvasculature and nanostructures within biocompatible hydrogels with submicron resolution. Projection micro-stereolithography (PμSL) and two-photon lithography offer high-speed, high-precision alternatives for creating master molds or direct printing of intricate scaffolds. The integration of sacrificial writing into functional tissue (SWIFT) and other embedded bioprinting techniques enables the fabrication of dense, vascularized tissues that more closely mimic renal parenchyma.

**Sensing and Data Acquisition: Towards Intelligent, Real-Time Monitoring.** The full potential of OoCs is unlocked by their ability to provide dynamic, quantitative readouts. However, the integration of miniaturized, non-invasive biosensors for real-time monitoring of key parameters (TEER, oxygen tension, specific biomarkers, pH) remains technically challenging. Most systems still rely on endpoint analyses, disrupting the culture and missing critical kinetic information. The subsequent challenge lies in managing, interpreting, and correlating the resulting multidimensional datasets from imaging, 'omics, and sensor streams. The path forward involves the co-development of embedded sensor technologies (e.g., optical sensor spots, graphene-based electrodes, plasmonic sensors) within microfluidic chips and advanced data science approaches. Leveraging machine learning and AI for the analysis of complex OoC data will be crucial for pattern recognition, predicting toxicity outcomes, and extracting biologically meaningful insights from high-content screens.

**Membrane Performance: Engineering the Ideal Filtration Interface.** For glomerular models, a central hurdle is fabricating a biomimetic membrane that concurrently fulfills multiple criteria: (1) Physiological thickness (<500 nm) to allow paracrine crosstalk; (2) Controlled pore size and distribution (2–8 nm) to mimic the molecular sieving properties of the GBM; (3) Surface charge to replicate charge selectivity; (4) Mechanical durability to withstand physiological pressures; and (5) Anti-fouling properties to prevent protein adhesion and cell overgrowth that occlude pores. Current track-etched and electrospun membranes often fall short in one or more of these aspects. Promising avenues include the development of ultrathin silicon nitride or graphene-based membranes with nanoscale precision, composite hydrogel membranes with tunable physical and chemical properties, and decellularized natural basement membrane scaffolds that provide a native-like biochemical milieu.

**Scalability and Standardization: The Bedrock of Translation and Adoption.** The field currently suffers from a lack of standardized testing protocols, manufacturing processes, and performance metrics, leading to poor inter-laboratory reproducibility and hindering direct comparison of results. Scaling systems for high-throughput screening in pharmaceutical applications introduces complexities in maintaining uniformity across dozens or hundreds of chips. To overcome this, a concerted effort is required to establish community-wide standards for cell sources, culture media, flow parameters, and benchmark compounds. Technologically, this will drive the adoption of injection-molded thermoplastics for mass production, microfluidic large-scale integration (mLSI) platforms, and automated imaging and liquid handling systems. Collaboration between academia, industry, and regulatory bodies, like Food and Drug Administration (FDA) and European Medicines Agency (EMA), is paramount to validate these platforms and define their role in the drug development and safety assessment pipeline.

In conclusion, while the path forward for renal barrier-on-a-chip technology is fraught with interconnected challenges, each presents a clear opportunity for transformative innovation. By adopting a multidisciplinary approach that converges advanced materials science, stem cell biology, high-precision engineering, data science, and regulatory science, the field can progress from creating fascinating proof-of-concept models to deploying robust, standardized, and clinically impactful tools. This evolution will ultimately reshape our approach to understanding kidney disease, developing safer pharmaceuticals, and realizing the promise of personalized medicine.

## 7 Summary

This review has comprehensively elaborated on the construction and evaluation of renal barrier biosystems based on OoC technology. The core of renal barrier OoC technology lies in the construction of highly simulated renal functional units in vitro through the multidisciplinary integration of microfluidic engineering, advanced biomaterials, and cell biology. Commencing from the biological basis of the renal barrier, we systematically elucidated the key structural and functional properties of the GFB, TRB, and CDRB, providing an indispensable biological blueprint for chip design.

Regarding the construction of renal barrier biochips, the focus was on how engineering approaches—encompassing material selection, precision fabrication, biomimetic structural design, and dynamic microenvironment simulation strategies—enable the reconstruction of these critical biological properties. For the evaluation of renal barrier models, a multidimensional assessment framework covering structural biomimicry, selective barrier permeability, dynamic transport and metabolism, and pathological responsiveness was summarized. This framework establishes the core validation criteria for model functional reliability.

In terms of application scenarios, the review highlighted the transformative value of this technology in overcoming the limitations of traditional models, advancing high-fidelity prediction of drug toxicity, elucidating mechanisms of human diseases, and fostering the development of innovative therapies. While challenges persist in material and membrane performance, fabrication and resolution constraints, sensing and data acquisition, and scalability and standardization, the ongoing optimization and standardization of the technology are expected to accelerate its deep translation into precision medicine and regenerative medicine, contributing significantly to the prevention and treatment of kidney diseases.

## CRediT authorship contribution statement

**Tuya Naren:** Writing – review & editing, Writing – original draft, Conceptualization. **Weikang Lv:** Writing – review & editing. **Abdellah Aazmi:** Writing – review & editing. **Yujun Wang:** Writing – review & editing. **Haoran Yu:** Writing – review & editing. **Jie Ying Lee:** Writing – review & editing. **Huixiang Yang:** Writing – review & editing. **Mengfei Yu:** Writing – review & editing. **Xiuxiu Jiang:** Writing – review & editing. **Huayong Yang:** Resources. **Liang Ma:** Writing – review & editing, Writing – original draft, Supervision, Resources, Funding acquisition, Conceptualization.

## Ethics approval and consent to participate

Not applicable.

## Declaration of interests

The authors declare no conflict of interest.
