# Supplementary material for: Construction, evaluation, and applications of renal barrier-on-a-chip system
Source: Bioact Mater. 2026 Jan 2;59:421–49. doi: 10.1016/j.bioactmat.2025.12.032 (PMC12805313; doi:10.1016/j.bioactmat.2025.12.032)
Supplement: Multimedia component 1 [file mmc1.docx]

| OoC | Organ-on-a-chip |
| --- | --- |
| GFB | Glomerular filtration barrier |
| TRB | Tubular reabsorption barrier |
| CDRB | Collecting duct regulatory barrier |
| 3D | Three-dimensional |
| 2D | Two-dimensional |
| SD | Slit diaphragm |
| ADH | Antidiuretic hormone |
| GBM | Glomerular basement membrane |
| PTECs | Proximal tubule epithelial cells |
| GMECs | Glomerular Microvascular Endothelial Cells |
| iPSCs | Induced pluripotent stem cells |
| DKD | Diabetic kidney disease |
| AKI | Acute kidney injury |
| CRISPR | Clustered Regularly Interspaced Short Palindromic Repeats |
| KIM-1 | Kidney injury molecule-1 |
| AQP2 | Aquaporin-2 |
| SGLT2 | Sodium-Glucose Cotransporter 2 |
| AGE | Advanced glycation end-product |
| PEG | Polyethylene glycol |
| PDMS | Polydimethylsiloxane |
| PMMA | Poly(methyl methacrylate) |
| PC | Polycarbonate |
| PET/PETE | Polyethylene terephthalate |
| COP | Cycloolefin polymer |
| PEGDA | Poly(ethylene glycol) diacrylate |
| VEGF | Vascular endothelial growth factor |
| ESCs | Embryonic stem cells |
| PK | Pharmacokinetics |
| GEnC | Glomerular endothelial cell |
| HUVECs | Human umbilical vein endothelial cells |
| TEER | Transepithelial/transendothelial electrical resistance |
| ECM | Extracellular matrix |
| TEM | Transmission electron microscopy |
| SEM | Scanning electron microscopy |
| CLSM | Confocal laser scanning microscopy |
| qPCR | Quantitative real-time polymerase chain reaction |
| ROS | Reactive oxygen species |
| OCR | Oxygen Consumption Rate |
| LDH | Lactate dehydrogenase |
| PKD | Polycystic kidney disease |
| FDA | Food and Drug Administration |
| EMA | European Medicines Agency |
| AI | Artificial intelligence |
| RGD | Arg-Gly-Asp |
